# Chemotherapy-elicited extracellular vesicle CXCL1 from dying cells promotes triple-negative breast cancer metastasis by activating TAM/PD-L1 signaling

**DOI:** 10.1186/s13046-024-03050-7

**Published:** 2024-04-23

**Authors:** Shengqi Wang, Jing Li, Shicui Hong, Neng Wang, Shang Xu, Bowen Yang, Yifeng Zheng, Juping Zhang, Bo Pan, Yudie Hu, Zhiyu Wang

**Affiliations:** 1 State Key Laboratory of Traditional Chinese Medicine Syndrome, State Key Laboratory of Dampness Syndrome of Chinese Medicine, The Second Affiliated Hospital of Guangzhou University of Chinese Medicine, Guangzhou, China; 2 The Research Center of Integrative Cancer Medicine, Discipline of Integrated Chinese and Western Medicine, The Second Clinical College of Guangzhou University of Chinese Medicine, Guangzhou, China; 3 Guangdong Provincial Key Laboratory of Clinical Research on Traditional Chinese Medicine Syndrome, Guangdong Provincial Academy of Chinese Medical Sciences, Guangdong Provincial Hospital of Chinese Medicine, Guangzhou, China; 4 Guangdong-Hong Kong-Macau Joint Lab on Chinese Medicine and Immune Disease Research, Guangzhou University of Chinese Medicine, Guangzhou, China; 5 The Research Center for Integrative Medicine, School of Basic Medical Sciences, Guangzhou University of Chinese Medicine, Guangzhou, China

**Keywords:** TNBC, Dying cell, Extracellular vesicle, CXCL1, Tumor-associated macrophage, PD-L1

## Abstract

**Background:**

Triple-negative breast cancer (TNBC) is the most aggressive subtype of breast cancer, and chemotherapy still serves as the cornerstone treatment functioning by inducing cytotoxic cell death. Notably, emerging evidence suggests that dying cell-released signals may induce cancer progression and metastasis by modulating the surrounding microenvironment. However, the underlying molecular mechanisms and targeting strategies are yet to be explored.

**Methods:**

Apoptotic TNBC cells induced by paclitaxel or adriamycin treatment were sorted and their released extracellular vesicles (EV-dead) were isolated from the cell supernatants. Chemokine array analysis was conducted to identify the crucial molecules in EV-dead. Zebrafish and mouse xenograft models were used to investigate the effect of EV-dead on TNBC progression in vivo.

**Results:**

It was demonstrated that EV-dead were phagocytized by macrophages and induced TNBC metastasis by promoting the infiltration of immunosuppressive PD-L1^+^ TAMs. Chemokine array identified CXCL1 as a crucial component in EV-dead to activate TAM/PD-L1 signaling. CXCL1 knockdown in EV-dead or macrophage depletion significantly inhibited EV-dead-induced TNBC growth and metastasis. Mechanistic investigations revealed that CXCL1^EV-dead^ enhanced TAM/PD-L1 signaling by transcriptionally activating EED-mediated PD-L1 promoter activity. More importantly, TPCA-1 (2-[(aminocarbonyl) amino]-5-(4-fluorophenyl)-3-thiophenecarboxamide) was screened as a promising inhibitor targeting CXCL1 signals in EVs to enhance paclitaxel chemosensitivity and limit TNBC metastasis without noticeable toxicities.

**Conclusions:**

Our results highlight CXCL1^EV-dead^ as a novel dying cell-released signal and provide TPCA-1 as a targeting candidate to improve TNBC prognosis.

**Graphical abstract:**

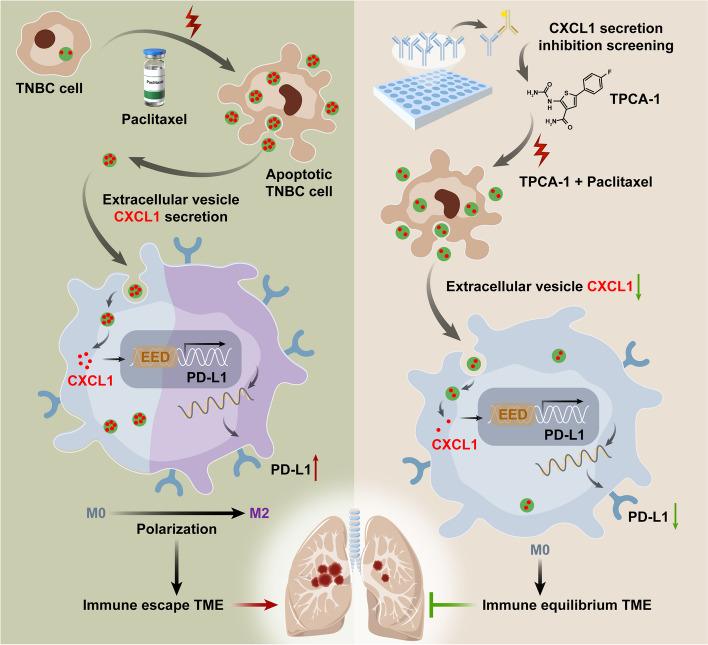

**Supplementary Information:**

The online version contains supplementary material available at 10.1186/s13046-024-03050-7.

## Background

Breast cancer is the most commonly diagnosed malignancy and the leading reason for cancer-associated mortality among women worldwide [1]. Breast cancer alone resulted in 2.3 million new cancer cases and 685,000 deaths in 2020 worldwide, accounting for 24.5% of new cancer cases and 15.5% of cancer deaths among women [1]. As the most malignant and aggressive subtype, TNBC comprises 15–20% of all breast cancers and is notorious for its early onset and poor prognosis, as well as short median overall survival (OS) once metastatic. Despite the significant advances in therapeutic strategies in recent decades, cytotoxic chemotherapy represents the cornerstone treatment for TNBC patients [2], especially the advanced or metastatic cases [3]. Chemotherapy substantially reduces the risk of death and distant recurrence in TNBC patients [4, 5]. However, its clinical effectiveness in TNBC patients is limited by the low response rate in some cases. For example, while the majority of TNBC patients undergo neoadjuvant chemotherapy, only approximately one-third of them achieve a pathologic complete response and experience favorable outcomes. In contrast, two-thirds of TNBC patients still have residual disease putting them at a high risk of relapse [2]. More importantly, chemotherapy often leads to secondary multidrug resistance, resulting in recurrence or metastasis. Notably, emerging studies have suggested that chemotherapy promotes breast cancer immune escape via stress-related machinery [6–9]. Therefore, it is important to further investigate the influence of chemotherapy on the biological behaviour of breast cancer cells and the underlying molecular mechanisms.

Cell death is a biological process that is fundamental to maintaining organismal homeostasis and defending against infection and cancer. It is estimated that more than 100 billion cells die and are renewed in the human body every day [10]. Dying cells are not simply inert cells waiting for removal, but instead, can release intracellular components as “goodbye” signals that actively modulate cellular fate in surrounding tissues [11]. Dying cell-released signals are multitudinous, and include metabolic molecules, cytokines, chemokines, proteins, nucleic acids, ion signals and various vesicles. Increasing evidence has indicated that dying cell-released signals are closely involved in cancer metabolism, angiogenesis, drug-resistance, metastasis, and tumor immunity [12, 13]. Dying cell-released signals mainly act as survival signals that promote the survival of surrounding cancer cells. Indeed, dying pancreatic cancer cells exhibit increased release of miR-194-5p, which promotes tumor survival and metastasis by activating the proliferation of residual tumor repopulating cells [13]. More importantly, dying cell-released signals often act as important messengers to regulate cancer immunogenicity, inflammatory cell infiltration, and immune response activity in the tumor microenvironment (TME) [14–16]. Dying cancer cells release numerous immunostimulatory damage-associated pattern molecules (DAMPs) as “find me” and “eat me” signals, which recruit and activate dendritic cells (DCs) or macrophages to trigger immune responses [14]. Dying cancer cells can also upregulate the expression and release of tumor-associated antigens and pro-inflammatory cytokines, leading to enhanced immune responses and improved immunotherapy outcomes [15, 16]. Increasing studies have indicated that chemotherapy might facilitate cancer immune escape and metastasis [6–9]. However, precise molecular mechanisms remain to be explored. Chemotherapy inevitably results in massive amounts of tumor cell death, leading to the release of multiple dying signals. Therefore, it is essential to investigate the roles and therapeutic implications of these dying signals in regulating cancer immune escape in the TME.

The TME plays a pivotal role in determining cancer chemoresistance and metastasis [17]. Accumulating studies have demonstrated that chemotherapy can alter the TME and enhance immune responses in the TME [18]. Tumor-associated macrophages (TAMs) are the major tumor-infiltrating immune cell population within multiple solid tumors including breast cancer [19]. Clinical evidence has revealed that TAM elevation usually predicates poor OS and clinical outcomes in patients with breast cancer [20]. TAMs are commonly regarded as the partners in crime of tumor cells, promoting tumor immune escape, angiogenesis, growth, and metastasis [19]. Notably, multiple chemotherapeutics, including paclitaxel, doxorubicin, and cyclophosphamide treatment, have been reported to stimulate breast cancer metastasis by generating a favorable pro-metastatic TME via increasing macrophage infiltration [6, 21]. However, the molecular mechanisms underlying chemotherapy-induced TAM elevation are still largely unknown. As the clearance of dying cancer cells and dying signals are mainly attributed to macrophage phagocytosis, it is important to investigate how macrophages respond to chemotherapy-induced dying signals to remodel the pro-metastatic TME.

Extracellular vesicles (EVs) are membrane-bound small vesicular bodies and are extracellularly secreted by exocytosis from cells. The content in EVs is heterogeneous, comprising proteins, DNAs, RNAs (mRNA, microRNA, and noncoding RNA), lipids, and metabolites [22]. EVs have emerged as an ideal tool for early diagnosis, prognostic prediction, and therapeutic drug delivery of various cancers owing to their natural ability to mediate intercellular communication, as well as their high stability, endogenous origin, and low immunogenicity [22, 23]. EVs play a central role in the TME by mediating intercellular communication between cancer and stromal cells [24]. Cancer cells use EVs as a novel mechanism to transfer the malignant phenotype to normal fibroblasts and endothelial cells to establish a pro-metastatic TME. In addition, tumor cell-derived EVs can inhibit the anti-cancer immune response by inducing the apoptosis of cytotoxic T cells, blocking the differentiation of monocytes into dendritic cells, and inducing the activation of immunosuppressive cells such as bone marrow suppressor cells (myeloid-derived suppressor cell, MDSC) and regulatory T cells (Tregs), which finally induce the immune escape of tumor cells [25–27]. Notably, almost all of the existing EV-related studies have focused on EVs released from living tumor cells [28, 29]. Chemotherapy will inevitably lead to massive cancer cell death and therefore increase the release of EVs from dying cancer cells. It has been reported the release of EVs during apoptosis is an active process. Cells usually release a significantly higher number of EVs upon induction of apoptosis [12]. To the best of our knowledge, the existing knowledge of the biological effect of dying cancer cell-released EVs on TME remodelling and tumor metastasis is limited. Keklikoglou et al. reported that neoadjuvant chemotherapy of breast cancer using taxanes and anthracyclines could elicit tumor-derived EVs with enhanced pro-metastatic capacity. These EVs could induce Ly6C^+^CCR2^+^ monocyte expansion in the pulmonary pre-metastatic niche to facilitate the establishment of lung metastasis [7]. Given the abundant infiltration and phagocytic nature of TAMs in the TME, it is interesting to investigate whether chemotherapy-induced dying cancer cell-released EVs could affect the immune escape and metastasis of TNBC by modulating TAMs in the TME.

In this study, we systematically demonstrated that the CXCL1^EV-dead^ signal, released from dying TNBC cells following chemotherapy, could favor TNBC immune escape and metastasis by transcriptionally activating TAM/PD-L1 signaling. In addition, TPCA-1 was screened as a promising small molecule to chemosensitize TNBC by inhibiting dying cell-released CXCL1^EV-dead^ signal.

## Materials and methods

### Cell culture and induction

Mouse TNBC 4 T1 cells (KG338) and Raw264.7 macrophages (KG240) were obtained from Nanjing KeyGen Biotech (Nanjing, China). Human acute monocytic leukemia cell line THP1 and TNBC cell line MDA-MB-231 were obtained from the American Type Culture Collection. 100 ng/ml phorbol-12-myristate-13-acetate (PMA, Sigma-Aldrich, Missouri, USA) was used to induce attachment and differentiation of THP1 monocytes into macrophages. 4 T1-Luc cells were generated by transfecting 4 T1 cells with the lentiviral luciferase reporter plasmid. For M1 and M2 macrophage induction, cells were stimulated with 100 ng/ml LPS, or 10 ng/ml IL-4 and 10 ng/ml IL-13 for 24 h, respectively. The identities of all these cell lines have been authenticated by short tandem repeat profiling.

### EV isolation, quantification, observation and particle size detection

For EV-dead isolation, TNBC cells were cultured in the EV-depleted medium and treated with 1 μM paclitaxel or 2 μM adriamycin (ADR) for 24 h to induce apoptosis. Then, cells were harvested and stained with annexin V-FITC solution (70-APCC101-100, MultiSciences, Hangzhou, China) for 5 min. The annexin V-FITC-positive cells were sorted by fluorescence-activated cell sorting technology using a FACS Aria III flow cytometer (BD Biosciences, Franklin Lakes, NJ, USA) and then cultured in the EV-depleted medium for 24 h. Then, the cell culture medium was harvested to isolate EV-dead using Ribo™ EV Isolation Reagent (C10130-2, Ribo Biotech, Guangzhou, China) or using a differential ultracentrifugation method [30]. Additionally, EV-alive was isolated from the cell culture medium of untreated TNBC cells. To investigate whether EVs derived from the annexin V^−^ 4 T1 cells can activate TAM/PD-L1 signaling, annexin V^−^ 4 T1 cells were sorted from both untreated and paclitaxel-treated 4 T1 cells. Subsequently, EVs were isolated from the conditioned medium (CM) of these sorted annexin V^−^ 4 T1 cells upon replating. During the isolation process of various EVs, a consistent number of sorted cells were replated. Subsequently, an identical volume of supernatant was collected for the isolation of EVs, which were resuspended in a uniform volume and subjected to assessment of size and concentration. These normalized approaches ensured the reliability of comparative analyses between samples. The protein concentration of EVs was quantified by the BCA method and the structure of EVs was observed by a transmission electron microscope [30]. EV sizes and numbers were detected by NTA using a Flow NanoAnalyzer (NanoFCM Inc., Xiamen, China) [31].

### Western blotting

Western blotting assay was conducted as previously reported [32]. The following antibodies were used: Alix (12422-1-AP, Proteintech, Wuhan, China), TSG101 (67381-1-Ig, Proteintech), CD81 (66866-1-Ig, Proteintech), Calnexin (ab133615, Abcam, Cambridge, MA, USA), iNOS (18985-1-AP, Proteintech), ARG1 (DF6657, Affinity, Changzhou, China), PD-L1 (DF6526, Affinity), PD-L1 (66248-1-IG, Proteintech), CXCL1 (AF5403, Affinity), EED (85,322 T, CST, MA, USA), and β-actin (4970S, CST).

### Zebrafish breast cancer xenotransplantation model

The zebrafish breast cancer xenotransplantation model was established as previously described [33]. Briefly, 200 Dil-stained 4 T1 cells were injected into the perivitelline space of each AB strain zebrafish embryo at 48 h post-fertilization using a microinjector. For the macrophage co-injection groups, 200 Dil-stained 4 T1 cells and 600 Raw264.7 cells were co-injected. For EV treatment or PD-L1 blockage, EVs and the anti-mouse PD-L1 monoclonal antibody (mAb) (BE0101-50 mg, BioXcell, Lebanon, NH, USA) were co-injected with cells at the indicated doses. After treatment for 48 h, breast cancer growth and metastasis in zebrafish were observed under a Nikon SMZ25 stereomicroscope.

### Animal experiments

Multiple batches of mouse 4 T1-Luc xenograft experiments were performed at different periods. For 4 T1-Luc xenograft establishment, 2 × 10^6^ 4 T1-Luc cells were inoculated subcutaneously into the mammary fat pads of female BALB/c mice (6 weeks old). The animals were randomly grouped using a random number table. To investigate the effect of EV-dead on TNBC growth and metastasis, 4 T1-Luc xenograft-bearing mice were randomly divided into the saline group (peritumoral injection with saline solution, 200 μl/20 g weight, q3d) and EV-dead group (peritumoral injection with EV-dead, 200 μg/20 g weight, q3d). To investigate the effect of EV-alive on TNBC growth and metastasis, 4 T1-Luc xenograft-bearing mice were randomly divided into the saline group and EV-alive group (peritumoral injection with EV-alive, 200 μg/20 g weight, q3d). To investigate the effect of CXCL1^EV-dead^ overexpression on TNBC growth and metastasis, 4 T1-Luc xenograft-bearing mice were randomly divided into three groups, including saline group, EV-dead group, and EV-dead^rCXC1^ group (peritumoral injection with EV-dead^rCXC1^, 200 μg/20 g weight, q3d). To investigate the effects of CXCL1^EV-dead^ knockdown or macrophage deletion on the pro-tumor activity of EV-dead, 4 T1-Luc xenograft-bearing mice were randomized into four groups, including saline group, EV-dead group, EV-dead^shCXCL1^ group (peritumoral injection with EV-dead^shCXC1^, 200 μg/20 g weight, q3d), and EV-dead + CL group (combined treatment with EV-dead and clodronate liposomes, CL). Subsequently, 200 μl CL (40337ES10, Yeasen Biotech, Shanghai, China) was injected intraperitoneally into 4 T1-Luc xenograft-bearing mice 2 days before the EV injection, following which, intraperitoneal injection (100 μl per mouse) was continued once every 2 weeks during the animal experiment period. To investigate the combined effect of TPCA-1 and paclitaxel treatment, 4 T1-Luc xenograft-bearing mice were randomly divided into four groups, including saline group, TPCA-1 group (10 mg/kg/d, intraperitoneal injection), paclitaxel group (10 mg/kg/3d, intraperitoneal injection), and TPCA-1 + paclitaxel group. To investigate the effect of TPCA-1 treatment on the pro-tumor activity of EV-dead, 4 T1-Luc xenograft-bearing mice were randomly divided into four groups: saline group, EV-dead group, EV-dead + TPCA-1 group, and EV-dead^rCXCL1^ + TPCA-L1 group. The administration doses of EV-dead, EV-dead^rCXCL1^, or TPCA-L1 were the same as those stated above. The mice were imaged using an IVIS Lumina XR in vivo imaging system (PerkinElmer, MA, USA) to monitor tumor growth and metastasis. According to international animal welfare recommendations, tumors that reached 20 mm in diameter were used as the experimental endpoint, at which mice were humanely euthanized. At the end of the animal experiment, mice were euthanized and the primary cells were isolated from tumors and subjected to macrophage phenotypic analysis as indicated below. The blood samples were subjected to biochemical analysis as previously reported [34] to detect the hepatotoxicity, nephrotoxicity, or hematotoxicity of TPCA-1, and HE staining assay was applied as previously reported [33] to investigate the lung metastasis differences in mice in different groups.

### Macrophage phenotype, population and PD-L1 expression analyses

For the phenotype analysis of Raw264.7 macrophages, cells were first treated as indicated. Then, Raw264.7 cells were harvested and incubated with FITC-conjugated F4/80 antibody (SC-71085, Santa Cruz, CA, USA), PE-conjugated CD206 antibody (141,705, Biolegend, CA, USA), or PE-Cy7-conjugated CD206 antibody (E-AB-F1135H, Elabscience, Houston, TX, USA) for 30 min at 37 °C. For the phenotype analysis of THP1 macrophages, cells were treated as indicated, harvested and incubated with APC-conjugated F4/80 antibody (17-4801-82, Invitrogen) and PE-conjugated CD163 antibody (12-1639-42, eBioscience) for 30 min at 37 °C. For the phenotypic analyses of primary macrophages isolated from mouse 4 T1-Luc xenografts, cells were incubated with CD45-PE-Cy7 (25-0451-82, eBioscience), F4/80-APC antibody (17-4801-82, Invitrogen), and CD206-PE antibody (141,705, Biolegend) for 30 min at 37 °C. For PD-L1 expression analyses of primary macrophages, cells were incubated with CD45-PE-Cy7 (25-0451-82, eBioscience), F4/80-FITC antibody (SC-71085, Santa Cruz), and PD-L1-APC antibody (124,312, Biolegend) for 30 min at 37 °C. After incubation, the cells were washed once with PBS and subjected to flow cytometry analysis.

### Macrophage phagocytosis assay

Briefly, EVs were labelled with the PKH67 green fluorescent cell linker (2 μM, MINI67, Sigma-Aldrich). The labelling process was stopped by adding serum to the mixture. Then, Raw264.7 cells were treated with PKH67-labeled EVs for 1–4 h. Red fluorescent polystyrene beads with an average particle size of 50 nm (PSRF00050, Zhongke Leiming Biotechnology, Beijing, China) were used as the negative control of EVs. After washing with PBS three times, Raw264.7 cells were harvested and subjected to flow cytometry to measure the green and red fluorescence intensities of the cells. To visualize the phagocytosis process of EVs by macrophages, the PKH67-treated macrophages were fixed, permeabilized, and then incubated with ActinRed (5 U/ml, KGMP0012, KeyGEN) for 20 min. Lastly, the cells were observed using an LSM710 confocal microscope (Zeiss, Oberkochen, Germany).

### Co-culture of TNBC cells and macrophages

The six- or 24-well Transwell co-culture system was used for the co-culture of TNBC cells and macrophages. In brief, Transwell inserts were placed in six- or 24-well culture plates. TNBC cells and macrophages were seeded into different Transwell chambers. The Transwell inserts were separated by a 0.4-μm permeable membrane that allowed the free exchange of media and soluble molecules. For the Transwell assay, an 8-μm pore size Transwell chamber was used to allow the migration of TNBC cells.

### Cell counting, wound healing, Transwell and CCK-8 assays

Cell counting assay was conducted [32] to investigate the proliferation ability of TNBC cells. Wound healing and Transwell assays were conducted [33] to investigate the migration and invasion abilities of TNBC cells when they were treated as indicated. CCK-8 assay was conducted [33] to investigate the viability of TNBC cells when they were treated with different EVs, TPCA-1, paclitaxel, or their combination.

### Cell apoptosis detection

Cell apoptosis of breast cancer cells after the indicated treatment was stained with annexin V-FITC/PI for 5 min using the Annexin V-FITC/PI Apoptosis Detection Kit (KGA107, KeyGEN BioTECH) according to the manufacturer’s protocol. Then, cell apoptosis was measured by the NovoCyte flow cytometer (ACEA Biosciences, CA, USA) or observed under a fluorescent microscope.

### Stem cell population analysis and in vitro limiting dilution assay

Stem cell population analysis was conducted [32] by detecting the ALDH^+^ subpopulation using the ALDEFLUOR Stem Cell Identification Kit (No.01700, STEMCELL, Cambridge, MA, USA), or by detecting the CD133^+^ subpopulation. Mouse CD133-PE (141,203, Biolegend) and human CD133-APC (372,806, Biolegend) antibodies were used. Flow cytometry detection was conducted using a NovoCyte flow cytometer (ACEA, Hangzhou, China), and analyzed using NovoExpress. The CD133^+^ breast cancer stem cells (BCSCs) were sorted using a FACS Aria III flow cytometer (BD Biosciences) and subjected to the in vitro limiting dilution assay as previously described [35]. Briefly, BCSCs were implanted into a 96-well plate at a gradient of 1, 5, 10, 20, 40 or 80 cells per well, and treated with the CM of untreated Raw264.7 cells or different EV-treated Raw264.7 cells. The ratio of tumourspheres was determined, and the sphere formation efficiency was calculated using the Extreme Limiting Dilution Analysis (http://bioinf.wehi.edu.au/software/elda) [36].

### Chemokine array assay

The Mouse Chemokine Array C1 (AAM-CHE-1-4, RayBio, Norcross, GA, USA) was used to analyze the chemokine composition differences between EV-alive and EV-dead. Briefly, the chemokine antibody-coated membranes were blocked with blocking buffer for 30 min and incubated with equal amounts of EV-alive or EV-dead solution overnight at 4 °C. Then, the membranes were washed and incubated with the biotin-conjugated detection antibody cocktail for 2 h, and the HRP-conjugated streptavidin for 1 h. Finally, the membranes were washed, subjected to chemiluminescence, developed, and photographed.

### CXCL1 secretion inhibitor screening

To screen the potential CXCL1 secretion inhibitor of 4 T1 cells from the Chemokine Inhibitor Library (L7600, TOPSCIENCE, Shanghai, China), 4 T1 cells were treated with 80 types of chemokine inhibitors (1 μM) for 48 h. Subsequently, the concentration of CXCL1 in cell culture supernatants was detected using the Mouse CXCL1 ELISA Kit.

### Elisa

An ELISA was conducted to detect the CXCL1 concentrations in different EV preparations. Briefly, equal quantities of EVs were lysed by RIPA and sonication. CXCL1 concentrations in the lysed EVs were detected using the CXCL1 ELISA Kit (USCN Business, Wuhan, China) as previously described [33].

### Immunofluorescence assay

Immunofluorescence analysis was conducted as previously described [32]. The following primary antibodies were used in the immunofluorescence assay including PD-L1 (DF6526, Affinity), CXCL1 (AF5403, Affinity), Flag (M185-3 L, MBL International Corporation, Woburn, MA, USA), CD206 (141,704, Biolegend), PD-L1 (66248-1-IG, Proteintech), EED (DF7308, Affinity), F4/80 (17-4801-82, Invitrogen, MA, USA), α-SMA (AF1032, Affinity), CD81 (66866-1-Ig, Proteintech) and CD206 (24,595 T, CST) antibodies. The following secondary antibodies were used in the immunofluorescence assay: Alexa Fluor® 555 conjugated-anti-rabbit IgG (4413S, CST), Alexa Fluor® 488 conjugated-anti-rabbit IgG (4412S, CST), Alexa Fluor® 555 conjugated-anti-mouse IgG (A21422, ThermoFisher, Waltham, MA, USA), FITC conjugated-anti-Rat IgG (SC-2011, Santa Cruz), Alexa Fluor® 488 conjugated-anti-mouse IgG (4408S, CST) and Alexa Fluor® 555 conjugated-anti-Rat IgG (4417S, CST). Fluorescence images were obtained using an LSM710 confocal microscope.

### Transfection of plasmid and siRNA

A commercialized CXCL1 recombinant plasmid with a C-terminal FLAG tag was purchased from Dahong Biosciences (Guangzhou, China). The shRNA plasmids for CXCL1, EED, and PD-L1 as well as the PD-L1 recombinant plasmid were purchased from Vigene Biosciences (Jinan, China). Plasmids were transfected into the indicated cells using the LipoFiter™ reagent (Hanbio Biotech, Shanghai, China) [32].

### Circulating tumor cell (CTC) detection

The number of CTCs in the blood of tumor-bearing mice was measured by detecting the relative expression levels of the luciferase gene derived from 4 T1-Luc cells. Genomic DNA was extracted from mouse peripheral blood and measured by QPCR assay using the following primers: 5′-GCTCAGCAAGGAGGTAGGTG-3′ (forward) and 5′-TCTTACCGGTGTCCAAGTCC-3′ (reverse) for luciferase; and 5′-GGAGGGGGTTGAGGTGTT-3′ (forward) and 5′-GTGTGCACTTTTATTGGTCTCAA-3′ (reverse) for mouse *β-actin*.

### QPCR

QPCR was conducted as previously described [32]. The primer sequences were as follows: 5′-GCTCCAAAGGACTTGTACGTG-3′ (forward) and 5′-TGATCTGAAGGGCAGCATTTC-3′ (reverse) for mouse *PD-L1*; 5′-GACTCCAGCCACACTCCAAC-3′ (forward) and 5′-TGACAGCGCAGCTCATTG-3′ (reverse) for mouse *CXCL1*; 5′-AGCCACCCTCTATTAGCAGTT-3′ (forward) and 5′-GCCACAAGAGTGTCTGTTTGGA-3′ (reverse) for mouse *EED*.

### Double luciferase reporter gene assay

The double luciferase reporter gene assay was conducted using the Secrete-Pair™ Dual Luminescence Assay Kit (LF031, Genecopeia, Rockville, MD, USA) [33] to investigate the *PD-L1* promoter activity changes of Raw264.7 cells when treated as indicated. The commercially available *PD-L1* promoter plasmid (MPRM25392-PG04, Genecopeia) was transfected into Raw264.7 cells using Vigenefection (FH880806, Vigene Biosciences) [33].

### DNA-pull down-MS

The DNA-pull down-MS assay was conducted by Huijun Biotechnology (Guangzhou, China) to identify the transcription factor responsible for the CXCL1-induced promoter activity of *PD-L1*. Briefly, the biotinylated promoter fragment of *PD-L1* was synthesized by PCR using the following primers: 5′-TCTTGAACGGCAAGACAAC-3′ (forward) and bio-5′-TTCTGACCCAGCTACCTAC-3′ (reverse). The pull-down experiments were conducted as previously described [37] using the biotinylated *PD-L1* promoter fragment. The purified proteins underwent MS analysis by Huijun Biotechnology. The enriched protein was obtained by comparing the identified proteins with the control group.

### Chromatin immunoprecipitation (CHIP)-PCR

CHIP assay was conducted by immune-precipitating the DNA fragments with EED antibody (85,322 T, CST) using the CHIP Assay Kit (P2078, Beyotime, Shanghai, China) [38]. Analysis of the genomic sequence of the *PD-L1* promoter (NC_000085.7:29342838-29,344,837) revealed a potential binding site (5′-GTTCCACTC-3′, site: − 437 to − 429 bp) for the transcription factor EED. This region in the immune-precipitated DNA samples was amplified by PCR assay using the following primers: 5′-AAGGTGGGAGCTGTAGAGGAA-3′ (forward) and 5′-TGCTACTGAGAGGCTGTCGAT-3′ (reverse).

### Statistical analysis

Student’s t-test and one-way ANOVA were used for comparisons among groups. Levene’s Test of Equality of Variances was used to assess the assumption of homogeneity of variance. Survival curves were calculated using Kaplan–Meier analysis and were compared using the log-rank test. Data are represented as the mean ± SD. Data with repeated measurements were analyzed by repeated-measures ANOVA followed by post-hoc test. All tests were 2-sided and *P* < 0.05 was considered statistically significant.

## Results

### EV-dead induces TNBC lung metastasis and promotes TAM infiltration in vivo

**Fig. 1:**
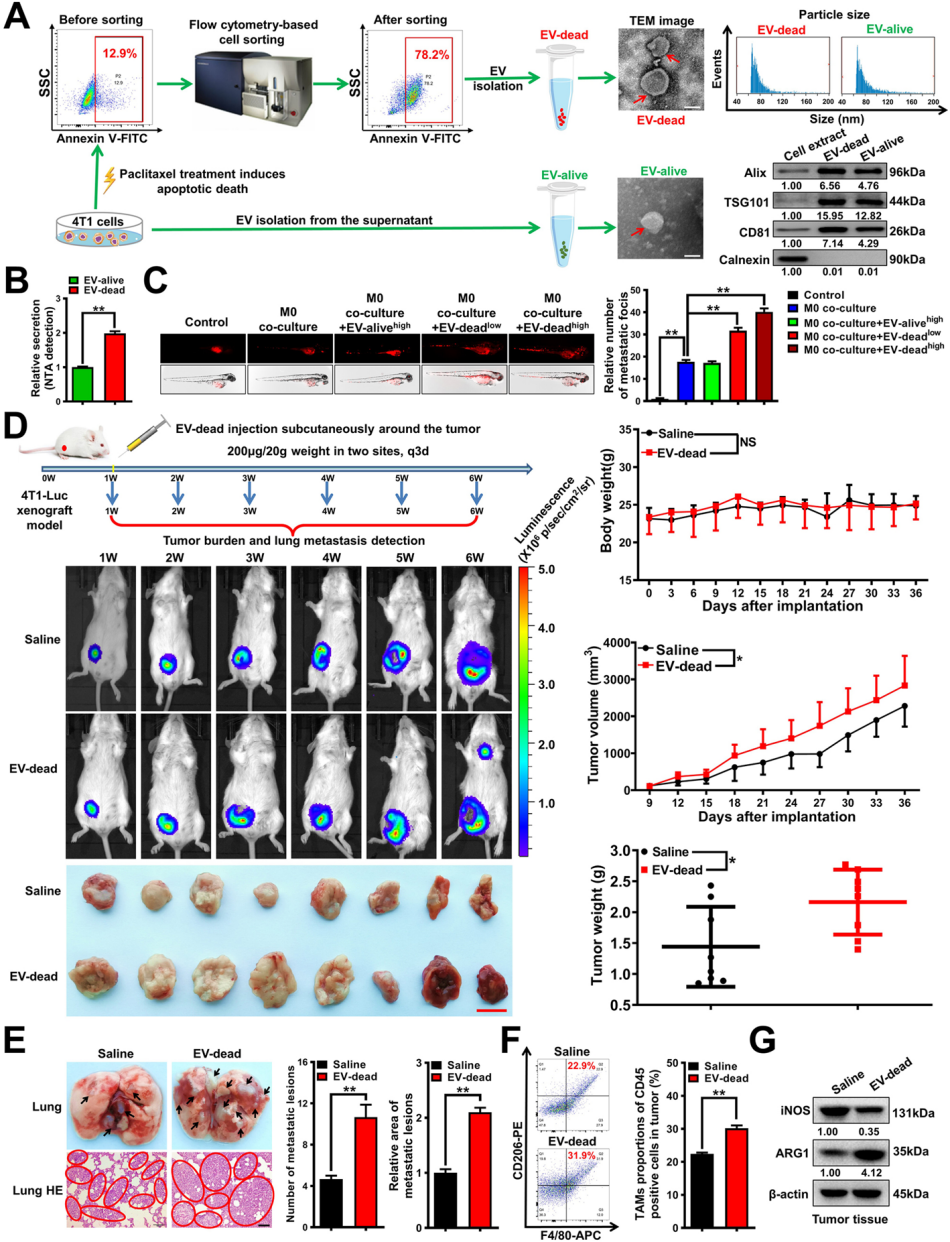
EV-dead induces TNBC lung metastasis and promotes TAM infiltration in vivo. **A** Diagram of the EV-dead and EV-alive separation procedures and their representative transmission electron microscopy (TEM) images. Scale bar: 100 nm. The sizes of the EVs were detected using a Flow NanoAnalyzer, and their protein markers were identified by Western blotting analysis. **B** NTA analysis was conducted to detect the relative secretion content of EV-dead and EV-alive; *n* = 3. **C** Representative images of zebrafish breast cancer xenotransplantation model assay. The effects of EV-alive (100 μg/ml) and EV-dead (50–100 μg/ml) on the metastasis of 4 T1 cells in the presence or absence of M0 co-injection were investigated; *n* = 6. **D** Schematic diagram of the animal assay and representative pictures of the in vivo imaging assay and tumors. Peritumoral injection with EV-dead (200 μg/20 g weight, q3d) promoted tumor growth of 4 T1-Luc xenografts, as evidenced by increases in both tumor volume and weight, but had no significant effect on the weight of the mice; *n* = 8. Scale bar: 1 cm. **E** Representative images of the lungs and the lung HE staining assay. Metastatic foci were identified by HE staining of the lung sections. Scale bar: 100 μm; *n* = 3. **F** Infiltration levels of CD45^+^/F4/80^+^/CD206^+^ TAMs in mammary tumors; *n* = 3. **G** Expression levels of ARG1 and iNOS in mammary tumor tissues; *n* = 3. Data are presented as mean ± SD. ^*^p < 0.05, ^**^p **< **0.01

Paclitaxel remains one of the most commonly used chemotherapeutics for TNBC treatment [39]. To isolate EV-dead, mouse TNBC 4 T1 cells were cultured in EV-depleted medium, and paclitaxel was used to induce apoptosis of 4 T1 cells. Subsequently, the annexin V^+^ apoptotic populations were sorted by flow cytometry. EV-dead and EV-alive were isolated from the supernatants of early apoptotic 4 T1 cells (Fig. S1) and untreated 4 T1 cells, respectively. For EV characterization, it was found that the isolated EVs exhibited a lipid bilayer structure and had a median diameter of 79.0 nm for EV-dead and 83.3 nm for EV-alive. Both EVs also exhibited elevated expression of EV-positive markers including Alix, TSG101, and CD81, while exhibiting little expression of the EV-negative marker calnexin (Fig. 1A). These results suggest the successful isolation of both EV-alive and EV-dead. Additionally, nanoparticle tracking analysis (NTA) results showed that paclitaxel treatment significantly elevated the number of EVs secreted from 4 T1 cells (Fig. 1B). Next, the effects of EV-dead and EV-alive on TNBC growth and metastasis were investigated in vitro and in vivo. Both EV-alive and EV-dead treatments had little effect on the proliferation of TNBC cells in vitro and in the zebrafish breast cancer xenotransplantation model in vivo (Fig. S2). Macrophages are considered as the most abundant immune cell subset in the TME of breast cancer [19]. Additionally, macrophages have a powerful ability to phagocytize foreign bodies, while phagocytosis also represents an efficient way for EV uptake. Interestingly, further investigations found that EV-dead (50–100 μg/ml) treatment significantly promoted the metastasis of 4 T1 cells in the presence of Raw264.7 macrophage co-injection, which was not significantly achieved by EV-alive (Fig. 1C). More importantly, peritumoral injection with EV-dead significantly promoted the growth and lung metastasis of mouse TNBC 4 T1-Luc xenografts (Fig. 1D, E), which was accompanied by the elevated infiltration of M2 phenotype macrophages in the TME (Fig. 1F, G). Meanwhile, EV-dead treatment had no significant effect on the body weights of mice (Fig. 1D). In contrast, EV-alive treatment exhibited no significant effect on the growth and lung metastasis of mouse 4 T1-Luc xenografts in vivo (Fig. S3). These results suggest that EV-dead may promote TNBC growth and metastasis by modulating macrophage polarization in the TME. Taken together, these findings show that dying TNBC cells-released EVs induce TNBC growth and lung metastasis, as well as elevating TAM infiltration in vivo.

### EV-dead promotes the metastasis and chemoresistance of TNBC cells by inducing macrophage M2 polarization

**Fig. 2:**
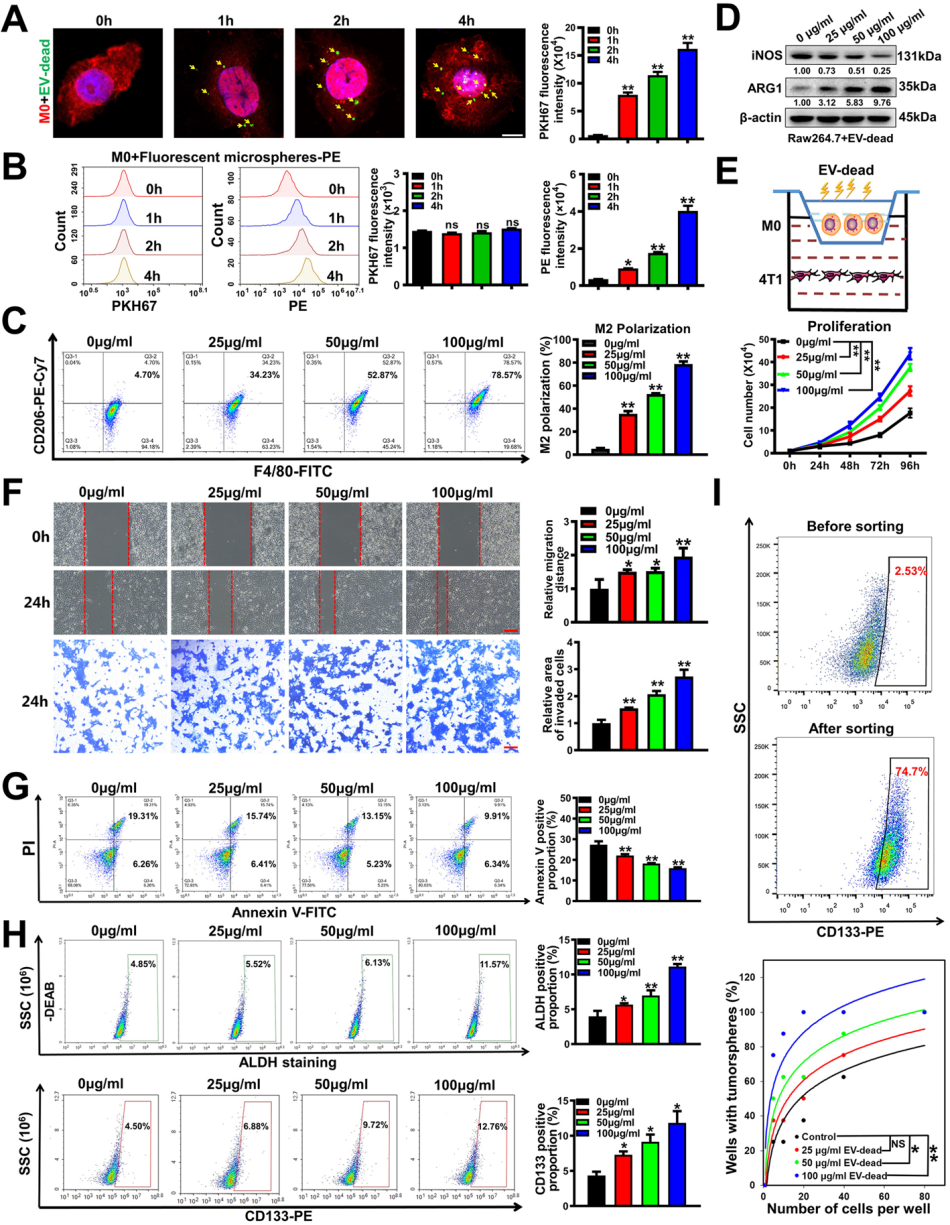
EV-dead promotes the metastasis and chemoresistance of TNBC cells by inducing macrophage M2 polarization. **A** EV-dead uptake by Raw264.7 macrophages was visualized using immunofluorescence labeling and quantified by flow cytometry. EV-dead was labeled with PKH67 (green). Raw264.7 macrophages were labeled with ActinRed (red) and DAPI (blue); Yellow arrows indicate EVs phagocytosed by Raw264.7 macrophages. *n* = 3. Scale bar: 5 μm. **B** The uptake of red fluorescent polystyrene beads (negative control of EV-dead) by Raw264.7 macrophages was quantified by flow cytometry. *n* = 3. **C**, **D** The polarization changes of Raw264.7 macrophages after EV-dead treatment (25–100 μg/ml) for 48 h; *n* = 3. **E** Diagram of 4 T1 and Raw264.7 cell co-culture using the Transwell system. Proliferation activity changes of the co-cultured 4 T1 cells after EV-dead treatment as indicated; *n* = 3. **F** Migration and invasion efficacy changes of the co-cultured 4 T1 cells after EV-dead treatment. Scale bars: 100 μm; *n* = 3. **G** Changes in apoptosis resistance of the co-cultured 4 T1 cells to paclitaxel after EV-dead treatment for 48 h, as determined by annexin V-FITC/PI staining and flow cytometry; *n* = 3. **H** The ALDH^+^ BCSC subpopulation and CD133^+^ BCSC subpopulation in 4 T1 cells after EV-dead treatment for 48 h were detected by flow cytometry. Diethylaminobenzaldehyde (DEAB) is a specific inhibitor of ALDH activity. *n* = 3. **I** The CD133^+^ BCSCs were sorted from 4 T1 cells, and the in vitro limiting dilution assay was conducted to further investigate the self-renewal activities of BCSCs after treatment with the CM of EV-dead-treated Raw264.7 cells; *n* = 8. Data are presented as mean ± SD. ^*^p < 0.05, ^**^p < 0.01

Next, we investigated whether EV-dead induces TNBC growth and metastasis by modulating TAMs in vitro. First, immunofluorescence and flow cytometry analysis showed that PKH67-labeled EV-dead could be time-dependently phagocytosed by Raw264.7 macrophages (Fig. 2A). As the negative control of EV-dead, the red fluorescent polystyrene beads (particle size, 50 nm) without cell membrane structure were also time-dependently phagocytosed by Raw264.7 macrophages, but did not cause the accumulation of cell membrane dye PKH67 in macrophages (Fig. 2B). These results suggest that PKH67 dye accumulation in macrophage is a result of EV uptake, but not accumulation from the residual dye. Undifferentiated macrophages (M0) can polarize into pro-inflammatory M1 phenotype or anti-inflammatory M2 phenotype under different stimulations. Flow cytometry and Western blotting results showed that EV-dead treatment significantly promoted M0 macrophage polarization into the M2 phenotype in a dose-dependent manner in vitro (Fig. 2C, D), whereas EV-alive exhibited no significant effect on that (Fig. S4A, B). Accumulating studies have suggested that TAMs can promote cancer growth and metastasis [40]. Therefore, TNBC 4 T1 cells and Raw264.7 macrophages were co-cultured in vitro using the Transwell model to simulate their coexistence. It was found that EV-dead administration (25–100 μg/ml) in the culture medium of Raw264.7 macrophages could significantly promote the proliferation (Fig. 2E), migration, and invasion abilities (Fig. 2F) of the co-cultured TNBC 4 T1 cells in a concentration-dependent manner. Additionally, EV-dead treatment promoted the apoptosis resistance of the co-cultured 4 T1 cells to paclitaxel (Fig. 2G). BCSCs are considered the root of breast cancer metastasis and chemoresistance [41]. EV-dead treatment also elevated both the ALDH^+^ BCSC subpopulation and the CD133^+^ BCSC subpopulation in 4 T1 cells (Fig. 2H). Additionally, in vitro limiting dilution assay showed that the CM of EV-dead treated Raw264.7 cells enhanced the self-renewal of the sorted CD133^+^ BCSCs (Fig. 2I). In contrast, EV-dead treatment showed no significant effects on the invasion, BCSC self-renewal, and chemosensitivity of the individually cultured 4 T1 cells. Moreover, EV-alive treatment also exhibited no significant effects on the invasion, BCSC self-renewal, and chemosensitivity of both individually cultured and co-cultured 4 T1 cells (Fig. S4C–F). Importantly, destroyed EV-dead could not induce the M2 polarization of Raw264.7 macrophages, or elevate the BCSC subpopulation of the co-cultured 4 T1 cells (Fig. S5).

**Fig. 3:**
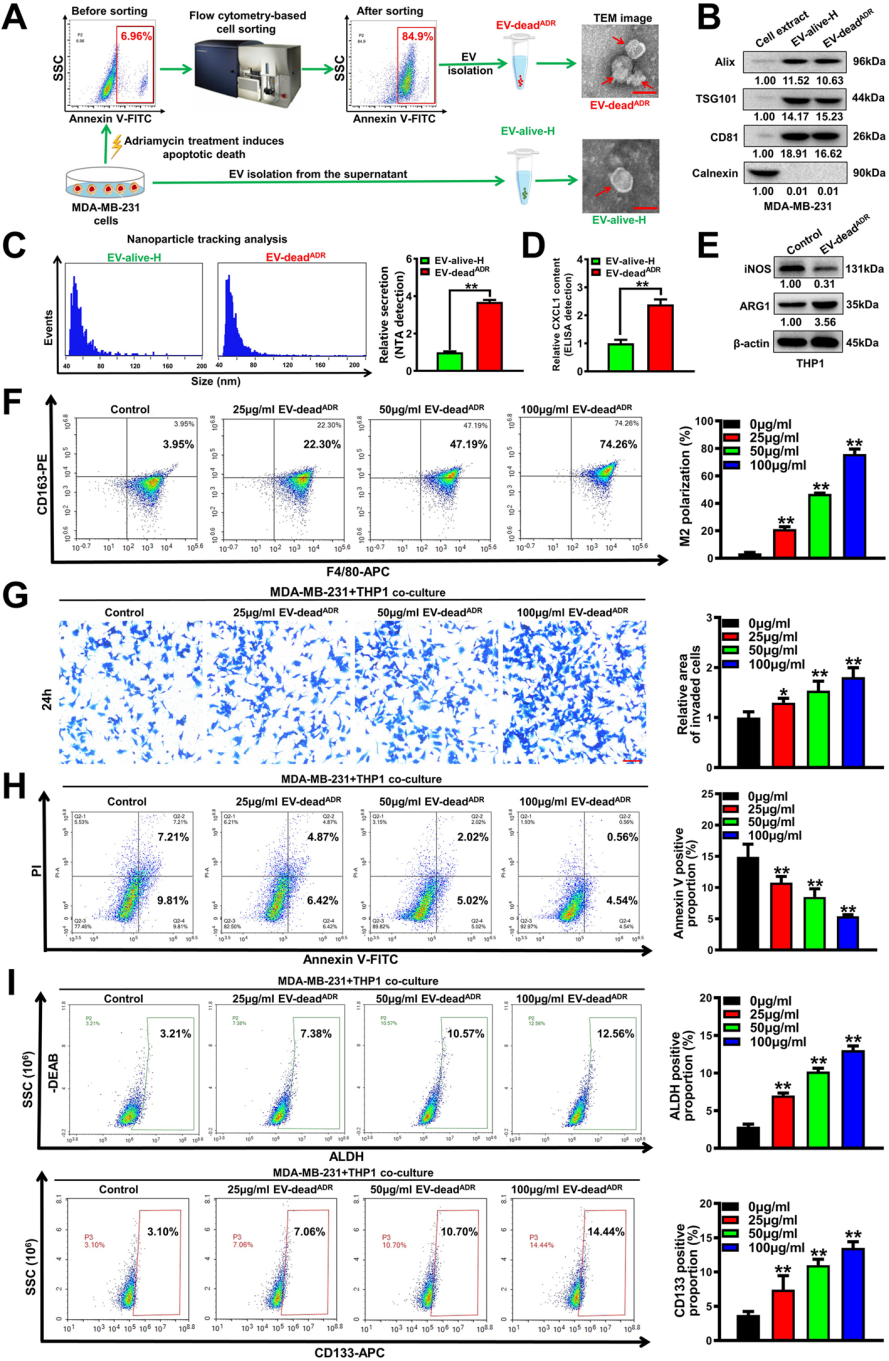
EV-dead promotes the invasion and BCSC subpopulation of the co-cultured human TNBC MDA-MB-231 cells by inducing macrophage M2 polarization. **A** Diagram of the EV-dead^ADR^ and EV-alive-H separation procedures and their representative TEM images. Scale bar: 100 nm. **B** EV protein markers were identified by Western blotting; (**C**) EV sizes and concentrations were detected by NTA analysis; (**D**) CXCL1 concentrations in EV-dead^ADR^ and EV-alive-H were detected by an ELISA assay; (**E**, **F**) The polarization changes of THP1 macrophages after EV-dead^ADR^ treatment (25–100 μg/ml) for 48 h; 50 μg/ml EV-dead was used for the Western blotting assay; **G** Invasion efficacy changes of the co-cultured MDA-MB-231 cells after EV-dead^ADR^ treatment as indicated. Scale bars: 100 μm; **H** Changes in apoptosis resistance of the co-cultured MDA-MB-231 cells to paclitaxel after EV-dead^ADR^ treatment for 48 h, as determined by annexin V-FITC/PI staining and flow cytometry; **I** The ALDH^+^ BCSC subpopulation and CD133^+^ BCSC subpopulation in the co-cultured MDA-MB-231 cells after EV-dead^ADR^ treatment for 48 h were detected by flow cytometry; *n* = 3. Data are presented as mean ± SD. ^*^p < 0.05, ^**^p < 0.01

To further validate the above finding, ADR, another classic chemotherapeutic drug in TNBC, was used to induce the apoptosis of the human TNBC cell line MDA-MB-231. Similarly, the early apoptotic populations were sorted by flow cytometry. ADR elicited EV-dead (EV-dead^ADR^) and the control EV-alive (EV-alive-H) were isolated from the supernatants of apoptotic human TNBC MDA-MB-231 cells and untreated MDA-MB-231 cells, respectively. TEM observation, immunoblotting assay and NTA analysis validated the successful isolation of these two kinds of EVs (Fig. 3A–C). It was found that ADR treatment significantly elevated the number of EVs secreted from 4 T1 cells (Fig. 3C). Furthermore, the ELISA assay indicated that EV-dead^ADR^ contained more CXCL1 than EV-alive of MDA-MB-231 cells (Fig. 3D). Moreover, EV-dead^ADR^ treatment also significantly induced the M2 polarization of human THP1 macrophages (Fig. 3E–F), and therefore promoted the invasion, apoptosis resistance and the BCSC subpopulation of MDA-MB-231 cells in the presence of THP1 macrophage co-culture (Fig. 3G–I). In contrast, EV-dead^ADR^ treatment exhibited little significant effects on the invasion and BCSC subpopulation of the individually cultured MDA-MB-231 cells. Additionally, EV-alive-H from MDA-MB-231 cells exhibited minimal significant effects on the M2 polarization of THP1 macrophages (Fig. S6A–B), as well as on the invasion and BCSC self-renewal of both individually cultured and co-cultured MDA-MB-231 cells (Fig. S6C–E). Importantly, destroyed EV-dead^ADR^ lost the induction effect on the M2 polarization of THP1 macrophages, and the self-renewal of BCSC subpopulation of the co-cultured MDA-MB-231 cells (Fig. S7). Taken together, these results indicate that EV-dead promotes the metastasis and chemoresistance of TNBC cells by inducing macrophage M2 polarization.

### CXCL1^EV-dead^ induces macrophage M2 polarization by activating PD-L1 expression

**Fig. 4:**
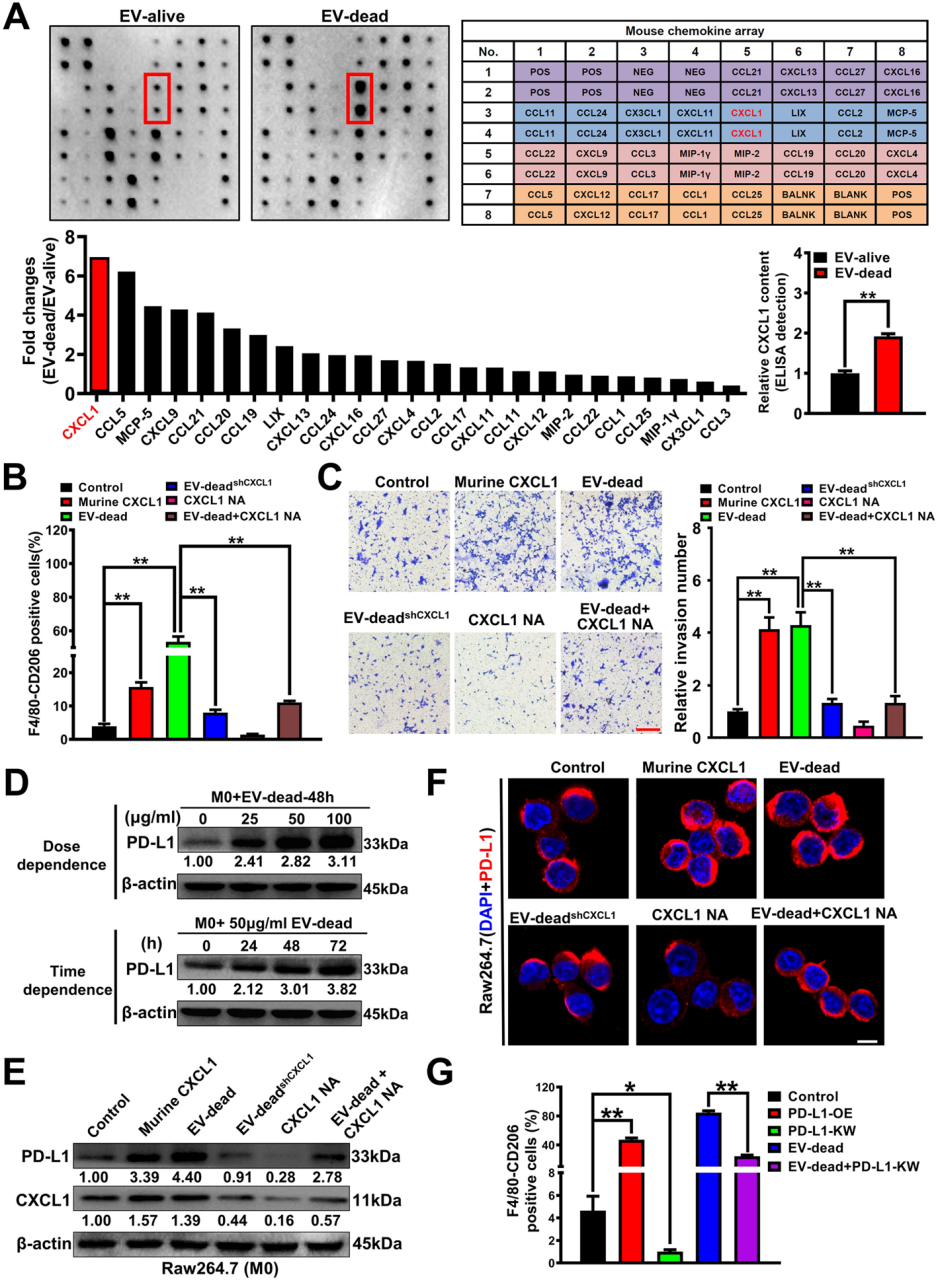
CXCL1^EV-dead^ induces macrophage M2 polarization by activating PD-L1 expression. **A** Chemokine array assay was conducted to characterize the differences in chemokine content between EV-dead and EV-alive. An ELISA assay was conducted to compare the relative CXCL1 content in EV-dead and EV-alive. **B** Changes in M2 phenotype polarization of Raw264.7 macrophages when treated with 10 ng/ml murine CXCL1, 50 μg/ml EV-dead, 50 μg/ml EV-dead^shCXCL1^, 5 μg/ml CXCL1 neutralizing antibody (NA), or EV-dead and CXCL1-NA combination for 48 h. **C** Representative images of Transwell assay. Raw264.7 macrophages were treated as indicated for 48 h and then co-cultured with 4 T1 cells. Scale bar: 200 μm. **D–F** Expression changes of CXCL1 and PD-L1 in Raw264.7 macrophages when treated as indicated for 48 h. Scale bar: 10 μm. **G** The results of the flow cytometry assay suggested that 50 μg/ml EV-dead treatment for 48 h induced the M2 polarization of Raw264.7 macrophages by activating PD-L1 expression; *n* = 3. Data are presented as mean ± SD. ^*^p < 0.05, ^**^p < 0.01

Next, we sought to determine the bioactive molecule in EV-dead that is responsible for inducing macrophage M2 polarization. As emerging evidence has suggested that chemokines play important roles in inducing the activation and polarization of macrophages [42], we aimed to characterize the abundant chemokines contained in EV-dead using a chemokine array. As shown in Fig. 4A, EV-dead contained multiple chemokines, among which, CXCL1 was the most abundant. More importantly, both the semiquantitative analysis of the chemokine array and quantitative ELISA confirmed that CXCL1 was significantly more upregulated in EV-dead compared to that in EV-alive. It has been reported that CXCL1 expression is significantly correlated with metastasis and poor OS in patients with breast cancer [43]. Therefore, we next investigated whether CXCL1 in EV-dead was responsible for the pro-metastatic effect of EV-dead. As shown in Fig. 4B, CXCL1 knockdown in EV-dead or CXCL1 neutralizing antibody (NA) partially abrogated the induction effect of EV-dead on the M2 polarization of macrophages, leading to the decreased invasion of TNBC cells in the co-culture system (Fig. 4C). Increasing studies have suggested that macrophages represent the major cellular source for maintaining PD-L1 expression in the TME, while PD-L1 is crucial for the activation and M2 polarization of macrophages [44, 45]. Here, Western blotting revealed that EV-dead could induce PD-L1 expression in macrophages in a time-and dose-dependent manner (Fig. 4D), while CXCL1 knockdown in EV-dead or CXCL1 NA administration inhibited PD-L1 expression (Fig. 4E, F). Meanwhile, it was found that EV-dead induced macrophage M2 polarization by activating PD-L1 expression, as shown by the finding that PD-L1 knockdown partially abrogated the M2 polarization of macrophages induced by EV-dead (Fig. 4G). Taken together, these findings show that CXCL1 is a crucial chemokine in EV-dead, which functions to mediate macrophage M2 polarization by activating PD-L1 expression. In contrast, EVs derived from the non-apoptotic annexin V^−^ 4 T1 cells were not enriched with CXCL1 expression and failed to activate TAM/PD-L1 signaling (Fig. S8).

### CXCL1^EV-dead^ promotes TNBC growth and lung metastasis in vivo by activating TAM/PD-L1 signaling

**Fig. 5:**
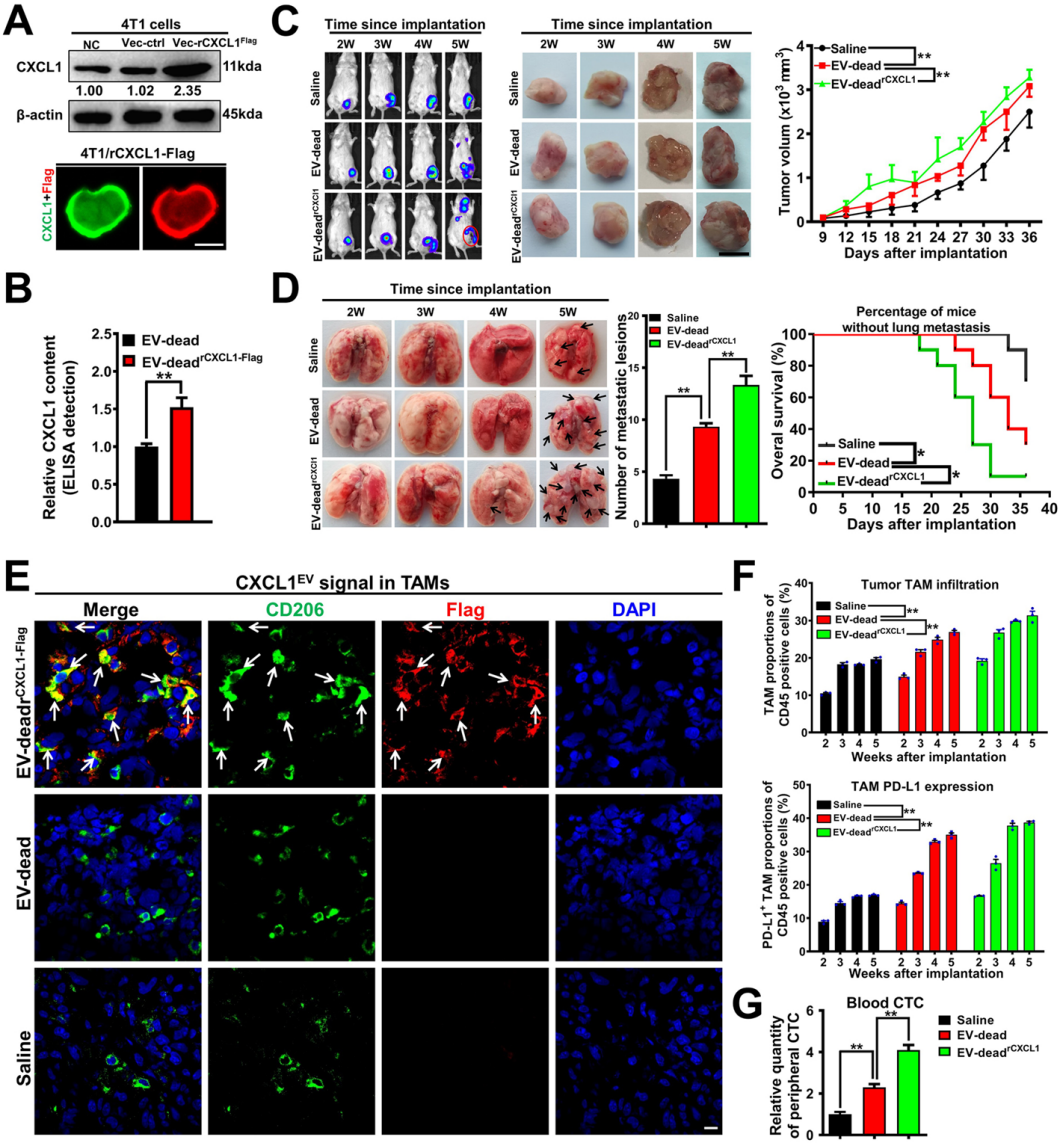
CXCL1^EV-dead^ promotes TNBC growth and lung metastasis in vivo by activating TAM/PD-L1 signaling. **A** The successful generation of 4 T1/rCXCL1^Flag^ cells was validated by immunoblotting and immunofluorescence assays. Scale bar: 5 μm. **B** The difference in CXCL1 content between EV-dead and EV-dead^rCXCL1-Flag^ was compared by ELISA. EV-dead^rCXCL1-Flag^ was isolated from the supernatants of apoptotic 4 T1/rCXCL1^Flag^ cells induced by paclitaxel treatment; *n* = 3. **C**, **D** Peritumoral injection with EV-dead^rCXCL1^ (200 μg/20 g weight, q3d) significantly accelerated TNBC growth (C) and lung metastasis (D) compared to that of the EV-dead group (200 μg/20 g weight, q3d); Tumor volume: *n* = 6; Number of metastatic lesions, *n* = 3; K-M curves of lung metastasis time, *n* = 10. Scale bar: 1 cm. **E** Tumor tissue immunofluorescence experiment showed that flag-tagged CXCL1 (red) from EV-dead^rCXCL1-Flag^ was predominantly phagocytosed by CD206^+^ macrophages (green) in the TME. Scale bar: 5 μm. **F** The infiltration levels of CD45^+^/F4/80^+^/CD206^+^ TAMs (up) and CD45^+^/F4/80^+^/PD-L1^+^ TAMs (down) in the TME of mice following treatment with saline, EV-dead, or EV-dead^rCXCL1-Flag^; *n* = 3. **G** QPCR assay was conducted to investigate the CTC quantity in the peripheral blood of mice following treatment with saline, EV-dead, or EV-dead^rCXCL1-Flag^; *n* = 3. Data are presented as mean ± SD. ^*^p < 0.05, ^**^p < 0.01

Next, we sought to validate whether CXCL1 in EV-dead is crucial in inducing TNBC growth and lung metastasis in vivo. To achieve this, Flag-tagged CXCL1 was overexpressed in 4 T1 cells and EV-dead^rCXCL1-Flag^ was isolated from the supernatants of apoptotic 4 T1/rCXCL1^Flag^ cells following paclitaxel treatment. As shown in Fig. 5A and Fig. S9A, immunoblotting, immunofluorescence and QPCR assays validated the successful generation of 4 T1/rCXCL1^Flag^ cells. Meanwhile, ELISA confirmed that CXCL1 was significantly more elevated in EV-dead^rCXCL1^ compared to EV-dead (Fig. 5B). It was also found that peritumoral injection with EV-dead^rCXCL1^ significantly accelerated the growth and lung metastasis of TNBC in the mouse 4 T1-Luc xenograft models compared to that of EV-dead (Fig. 5C, D), which also resulted in decreased OS of the mammary tumor-bearing mice. More importantly, the tumor tissue immunofluorescence experiment clearly showed that Flag-tagged CXCL1 was predominantly phagocytosed by CD206^+^ TAMs in the TME (Fig. 5E), which promoted PD-L1 expression and subsequent M2 polarization (Fig. 5E, F). CTCs are a rare population of tumor cells that contribute to the development of metastatic disease following their release into the peripheral circulation from primary tumor sites. In this study, peripheral CTCs were detected by QPCR analysis using primers directed to the luciferase genes of 4 T1-Luc cells. It was found that EV-dead^rCXCL1^ injection increased CTCs by 4.1-fold while EV-dead injection elevated CTCs by 2.3-fold (Fig. 5G), suggesting an increased metastatic potential of TNBC cells induced by EV-dead^rCXCL1^ compared to EV-dead. These findings indicate that CXCL1^EV-dead^ promotes the growth and lung metastasis of TNBC by activating TAM/PD-L1 signaling. Notably, PD-L1 blockage using an anti-PD-L1 mAb partially abrogated the induction effect of CXCL1^EV-dead^ on the proliferation and invasion of the co-cultured 4 T1 cells in vitro. Importantly, PD-L1 blockage also partially attenuated the promotion effect of CXCL1^EV-dead^ on the proliferation and metastasis of the co-cultured 4 T1 cells in the zebrafish breast cancer xenotransplantation model in vivo (Fig. S9B–D).

### CXCL1 knockdown in EV-dead or macrophage depletion inhibits EV-dead-induced TNBC growth and lung metastasis in vivo

**Fig. 6:**
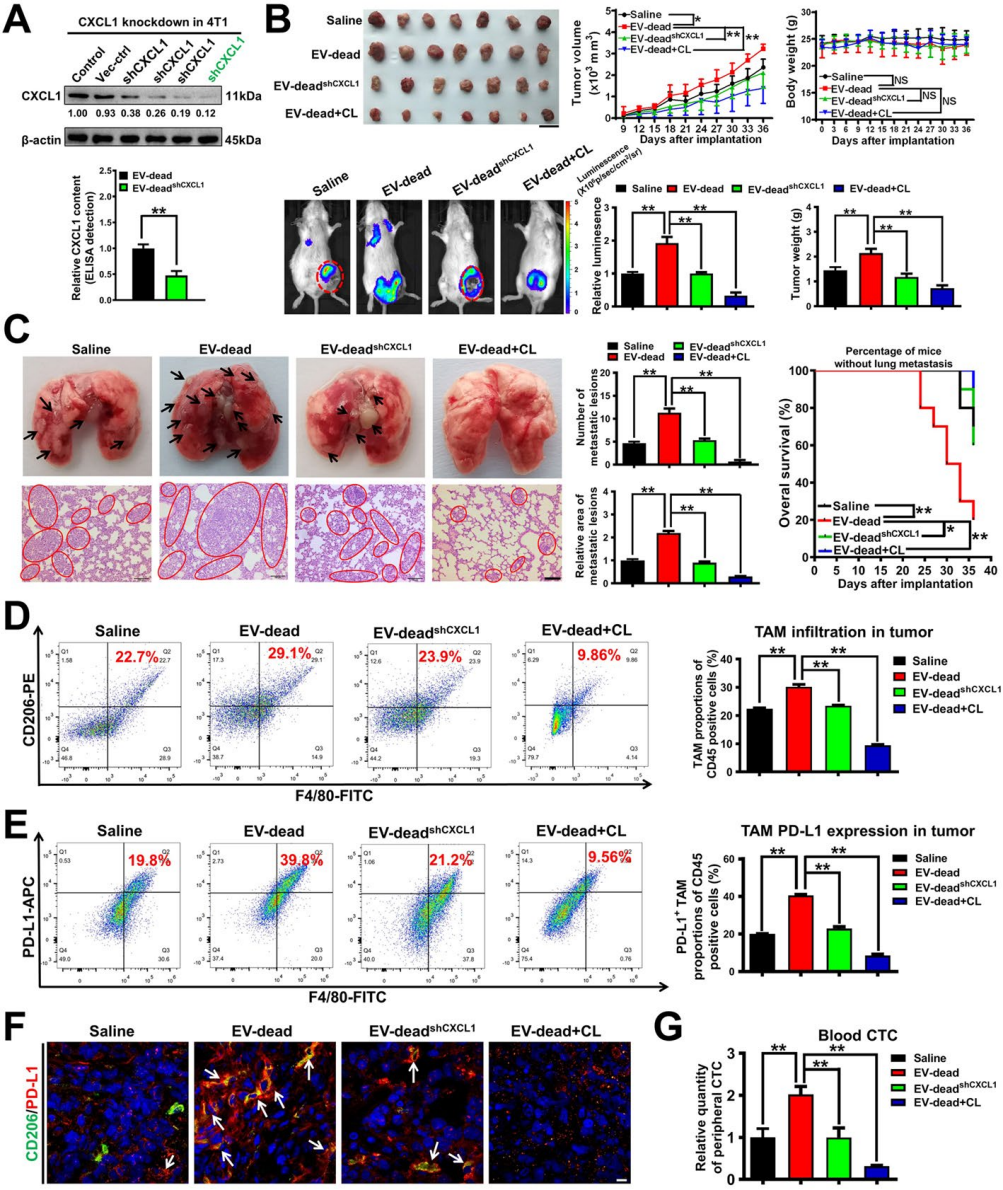
CXCL1 knockdown in EV-dead or macrophage depletion inhibits EV-dead-induced TNBC growth and lung metastasis in vivo*.* **A** The successful generation of 4 T1/shCXCL1 cells was verified by western blotting assay. The difference in CXCL1 content between EV-dead and EV-dead^shCXCL1^ was compared by ELISA. EV-dead^shCXCL1^ was isolated from the supernatants of apoptotic 4 T1/shCXCL1 cells induced by paclitaxel treatment; *n* = 3. **B** Representative images of the tumors (*n* = 7) and the in vivo imaging assay (*n* = 3), and mouse weight and tumor volume curves (*n* = 7). Clodronate liposomes (CL) were used to deplete macrophages in the TME of the 4 T1-Luc xenograft model. Scale bar: 2 cm. **C** Representative images of the lungs (*n* = 3) and lung HE assay (*n* = 3) as well as the K-M curves of lung metastasis time (*n* = 10). Scale bar: 100 μm. **D**, **E** The infiltration levels of CD45^+^/F4/80^+^/CD206^+^ TAMs (D) and CD45^+^/F4/80^+^/PD-L1^+^ TAMs (E) in the TME of mice following treatment with EV-dead, EV-dead^shCXCL1^, or the combination of EV-dead and CL; *n* = 3. **F** CD206 (green) and PD-L1 (red) expression levels in the TME. Arrows indicate PD-L1 expression in TAMs. Scale bar: 10 μm. **G** The quantity of CTCs in the peripheral blood of mice treated as indicated; *n* = 3. Data are presented as mean ± SD. ^*^p < 0.05, ^**^p < 0.01

Next, CXCL1 knockdown in EV-dead or macrophage depletion in the TME was applied to further validate the crucial roles of CXCL1 and TAMs in EV-dead-induced TNBC growth and lung metastasis. To achieve this, EV-dead^shCXCL1^ was isolated from the supernatants of apoptotic 4 T1/shCXCL1 cells induced by paclitaxel treatment. ELISA showed that the CXCL1 level in EV-dead^shCXCL1^ was significantly downregulated compared to that in EV-dead (Fig. 6A and Fig. S10A). Macrophages in the TME were specifically depleted by CL treatment. As shown in Fig. 6B–C, both CXCL1 knockdown in EV-dead and macrophage depletion partially inhibited the promotion effect of EV-dead on TNBC growth and lung metastasis in the mouse 4 T1-Luc xenograft model, leading to the increased OS of the mammary tumor-bearing mice. Meanwhile, both CXCL1 knockdown in EV-dead and macrophage depletion remarkably decreased the intratumoral infiltration levels of CD45^+^/F4/80^+^/CD206^+^ TAMs (Fig. 6D) and CD45^+^/F4/80^+^/PD-L1^+^ TAMs induced by EV-dead (Fig. 6E, F), which was also accompanied by decreased CTCs in the blood of 4 T1-Luc xenograft-bearing mice (Fig. 6G). These results indicated that CXCL1^EV-dead^ played an important role in recruiting TAMs and activating their PD-L1 expression. As shown in Fig. S10B, EV-dead treatment exhibited little significant effect on the expression of α-SMA (fibroblast marker), whereas CL addition significantly decreased α-SMA expression in mouse mammary tumor tissue. Taken together, these findings demonstrate that CXCL1 is the crucial bioactive chemokine in EV-dead to induce TNBC growth and metastasis, while macrophages but not fibroblasts act as the essential target cells of EV-dead in this biological process. This may be attributed to that EV-dead was predominantly phagocytosed by CD206^+^ TAMs in the TME.

### CXCL1^EV-dead^ transcriptionally increases PD-L1 expression in macrophages by activating EED signaling

**Fig. 7:**
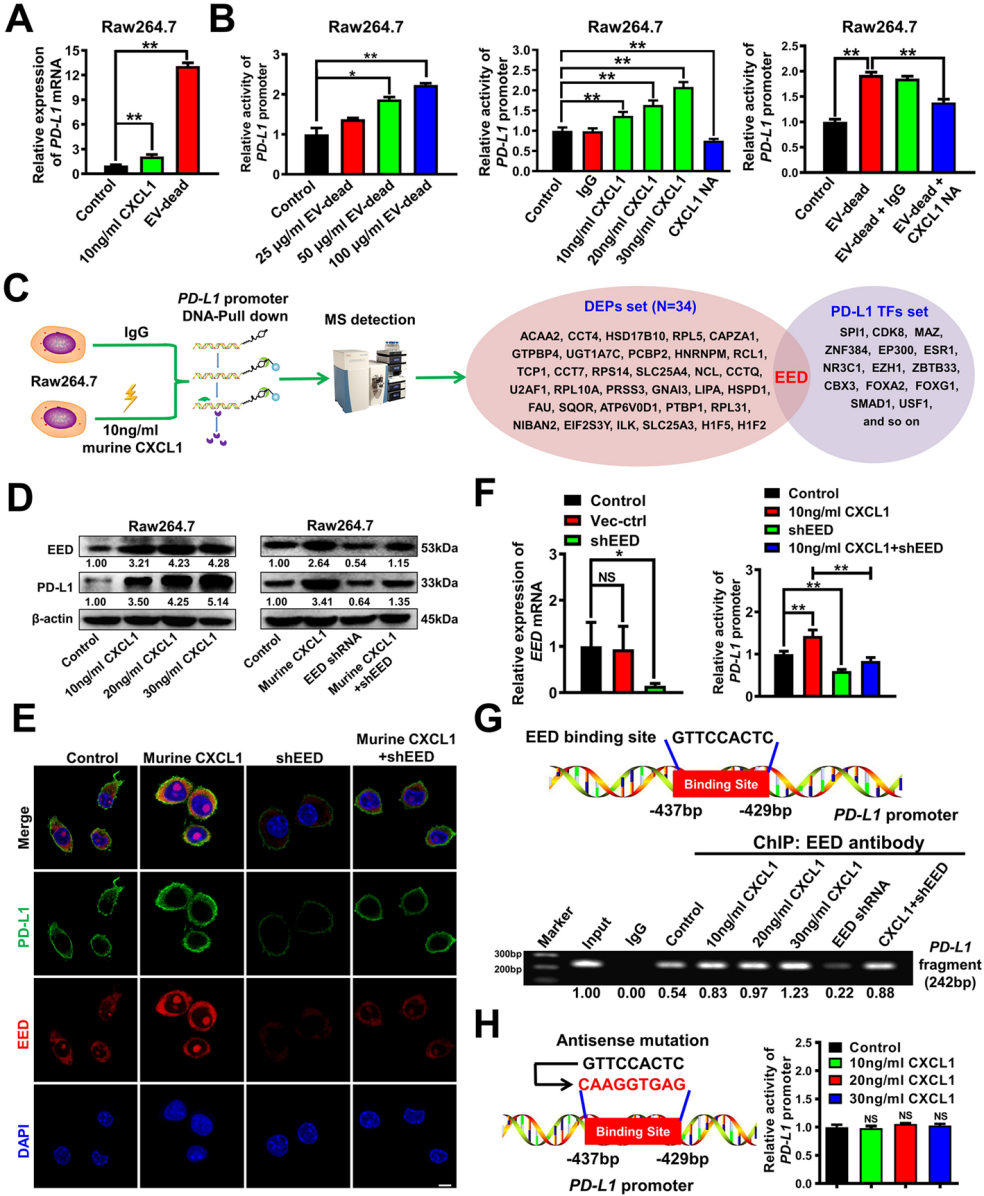
CXCL1^EV-dead^ transcriptionally increases PD-L1 expression in macrophages by activating EED signaling. **A** The mRNA level of *PD-L1* in Raw264.7 macrophages was significantly increased by 10 ng/ml murine CXCL1 and 50 μg/ml EV-dead treatment for 24 h; (**B**) The promoter activity of *PD-L1* in Raw264.7 cells when treated as indicated for 24 h; CXCL1-NA concentration: 5 μg/ml; (**C**) Diagram of the DNA-pull down-MS assay. The DEPs that bind with the *PD-L1* promoter region of Raw264.7 macrophages after CXCL1 treatment for 24 h were analyzed by mass spectrometry. The TFs of *PD-L1* were predicted using the hTFtarget database. EED was selected as the potential transcription factor by taking the intersection of the DEP set and the TF set. **D**, **E** The expression levels of EED and PD-L1 in Raw264.7 cells when treated as indicated for 48 h. Murine CXCL1 concentration: 10 ng/ml. Scale bar: 5 μm. **F** The combinational effect of CXCL1 treatment and EED knockdown on the promoter activity of *PD-L1* in Raw264.7 cells when treated as indicated for 24 h; (**G**) The binding activity of EED with the promoter fragment of *PD-L1* in Raw264.7 cells when treated as indicated for 48 h was investigated by CHIP-PCR assay. **H** The antisense mutation of the EED binding region in the *PD-L1* promoter significantly abrogated the induction effect of CXCL1 on *PD-L1* promoter activity in Raw264.7 macrophages; *n* = 3. Data are presented as mean ± SD. ^*^p < 0.05, ^**^p < 0.01

Next, we sought to investigate the molecular mechanism by which CXCL1^EV-dead^ elevated PD-L1 expression in macrophages. Similar to the function of recombinant murine CXCL1, EV-dead treatment significantly increased the mRNA level of *PD-L1* in Raw264.7 macrophages (Fig. 7A). Additionally, both recombinant murine CXCL1 and EV-dead could concentration-dependently activate the *PD-L1* promoter activity in Raw264.7 macrophages, which was partially inhibited by CXCL1 NA administration (Fig. 7B). These results indicated that CXCL1^EV-dead^ transcriptionally induced PD-L1 expression in Raw264.7 macrophages. To characterize the regulators involved in CXCL1-induced *PD-L1* transcription activation, DNA-pull down-MS assay was conducted to detect the deferentially expressed proteins (DEPs) binding with the *PD-L1* promoter region of Raw264.7 macrophages following CXCL1 treatment. The pull-down assay was conducted using the biotin-labelled *PD-L1* promoter fragment while the *PD-L1* promoter-interacting proteins were identified by mass spectrometry. A total of 34 DEPs were identified after CXCL1 treatment. Additionally, the potential transcription factors (TFs) of *PD-L1* gene were predicted using the hTFtarget database. By intersecting the DEP set and the TF set, embryonic ectoderm development protein (EED) was finally determined as the only common factor (Fig. 7C). Next, the combined effect of CXCL1 and EED knockdown on PD-L1 expression in macrophages was further investigated to validate whether EED was the potential transcription factor responsible for CXCL1-induced *PD-L1* transcription. CXCL1 significantly induced the expression and nuclear translocation of EED, and therefore activated the promoter activity and protein expression of PD-L1 in Raw264.7 macrophages (Fig. 7D–F). However, EED knockdown partially abrogated CXCL1-induced PD-L1 transcription and protein expression (Fig. 7E, F). Next, the binding sites of EED in the *PD-L1* promoter region as well as the binding activity changes after CXCL1 treatment were further investigated to better elucidate the molecular mechanism. JASPAR prediction suggested that there was one potential EED binding site (5′-GTTCCACTC-3′, − 437 to − 429 bp) in the *PD-L1* promoter region. CHIP-PCR assay further suggested that CXCL1 significantly promoted the binding of EED with this promoter fragment, while EED knockdown in macrophages partially abrogated their interaction (Fig. 7G). More importantly, the antisense mutation of the EED binding region in the *PD-L1* promoter dramatically abrogated the induction effect of CXCL1 on *PD-L1* promoter activity in Raw264.7 macrophages (Fig. 7H). Taken together, these results indicate that CXCL1^EV-dead^ transcriptionally increases PD-L1 expression in macrophages by activating EED signaling.

### TPCA-1 significantly inhibits CXCL1^EV-dead^-induced chemoresistance and invasion of TNBC cells co-cultured with macrophages

**Fig. 8:**
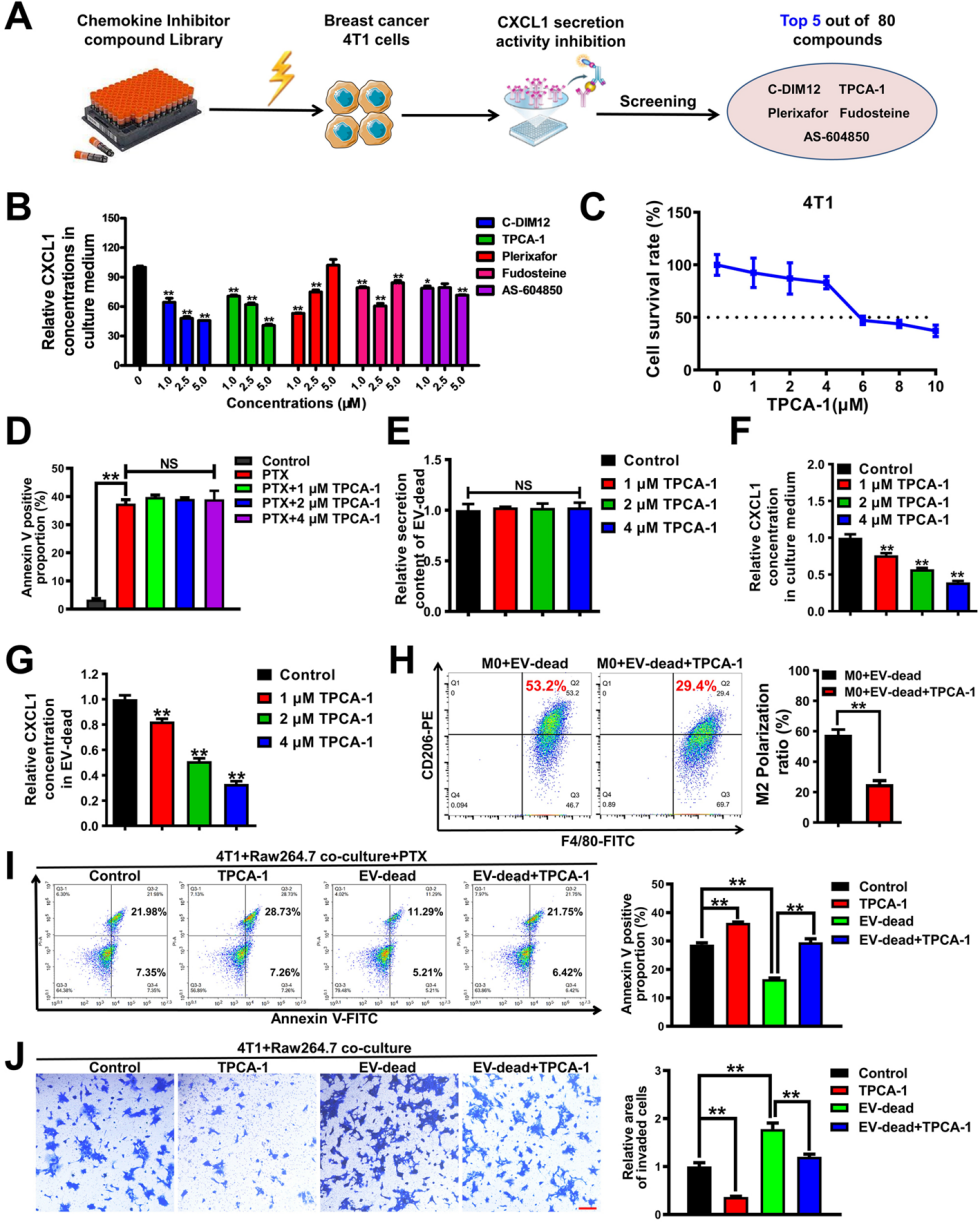
TPCA-1 significantly inhibits CXCL1^EV-dead^-EV-dead-induced chemoresistance and invasion of TNBC cells co-cultured with macrophages. **A**, **B** Diagram of the CXCL1 secretion inhibitor screening assay. 4 T1 cells were treated with 80 kinds of compounds (1 μM) for 48 h, and the top five compounds with the strongest inhibitory activities on CXCL1 secretion of 4 T1 cells were selected; *n* = 3. **C**, **D** The cytotoxicity of TPCA-1 in TNBC 4 T1 cells (*n* = 8) and its effect on the apoptosis resistance of 4 T1 cells to paclitaxel (*n* = 3). Cells were treated as indicated for 48 h. **E–G** TPCA-1 treatment for 48 h had no significant effect on EV-dead secretion (**E**) from 4 T1 cells but significantly attenuated the concentration of CXCL1 in the supernatants of 4 T1 cells (**F**) and the EV-dead of apoptotic 4 T1 cells (**G**); *n* = 3. **H** The results of the flow cytometry assay indicated that 1 μM TPCA-1 treatment for 48 h significantly reversed the induction effect of EV-dead (50 μg/ml) on the M2 polarization of macrophages; *n* = 3. **I, J** Apoptosis and Transwell assays suggested that TPCA-1 treatment (1 μM) for 48 h inhibited 50 μg/ml EV-dead-induced apoptosis resistance (*n* = 3) and invasion (*n* = 3) of 4 T1 cells in the co-culture system; Scale bar: 100 μm. Data are presented as mean ± SD. ^*^p < 0.05, ^**^p < 0.01

Next, we explored the translational significance of targeting CXCL1^EV-dead^ signaling. As CXCL1 is a chemokine, the commercialized Chemokine Inhibitor Compound Library (TargetMol, Catalog Number: L7600) containing 80 small molecule compounds was screened by ELISA (Fig. 8A). The top five compounds with the strongest inhibitory activities on CXCL1 secretion from TNBC cells were identified as C-DIM12, TPCA-1, plerixafor, fudosteine, and AS-604850. TPCA-1 was selected and subjected to further investigations given that it dose-dependently inhibited CXCL1 secretion in 4 T1 cells significantly (Table S1 and Fig. 8B). At concentrations of 1–4 μM, TPCA-1 exhibited little cytotoxicity effects on 4 T1 cells (Fig. 8C), and did not affect the apoptosis resistance of 4 T1 cells to paclitaxel (Fig. 8D and Fig. S11). Additionally, TPCA-1 had minimal significant effect on EV-dead secretion from 4 T1 cells (Fig. 8E) but significantly inhibited CXCL1 levels in both the supernatants of 4 T1 cells (Fig. 8F) and the EV-dead of apoptotic 4 T1 cells (Fig. 8G). More importantly, TPCA-1 remarkably reversed the induction effect of EV-dead on macrophage M2 polarization (Fig. 8H), which suppressed the EV-dead-induced apoptosis resistance (Fig. 8I) and invasion (Fig. 8J) of TNBC cells in the co-culture system. Taken together, these results identify TPCA-1 as an inhibitor of CXCL1^EV-dead^ secretion, which can significantly suppress the CXCL1^EV-dead^-induced chemoresistance and invasion of TNBC cells co-cultured with macrophages.

### TPCA-1 chemosensitizes TNBC to paclitaxel and inhibits CXCL1^EV-dead^-induced TNBC growth and lung metastasis in vivo

**Fig. 9:**
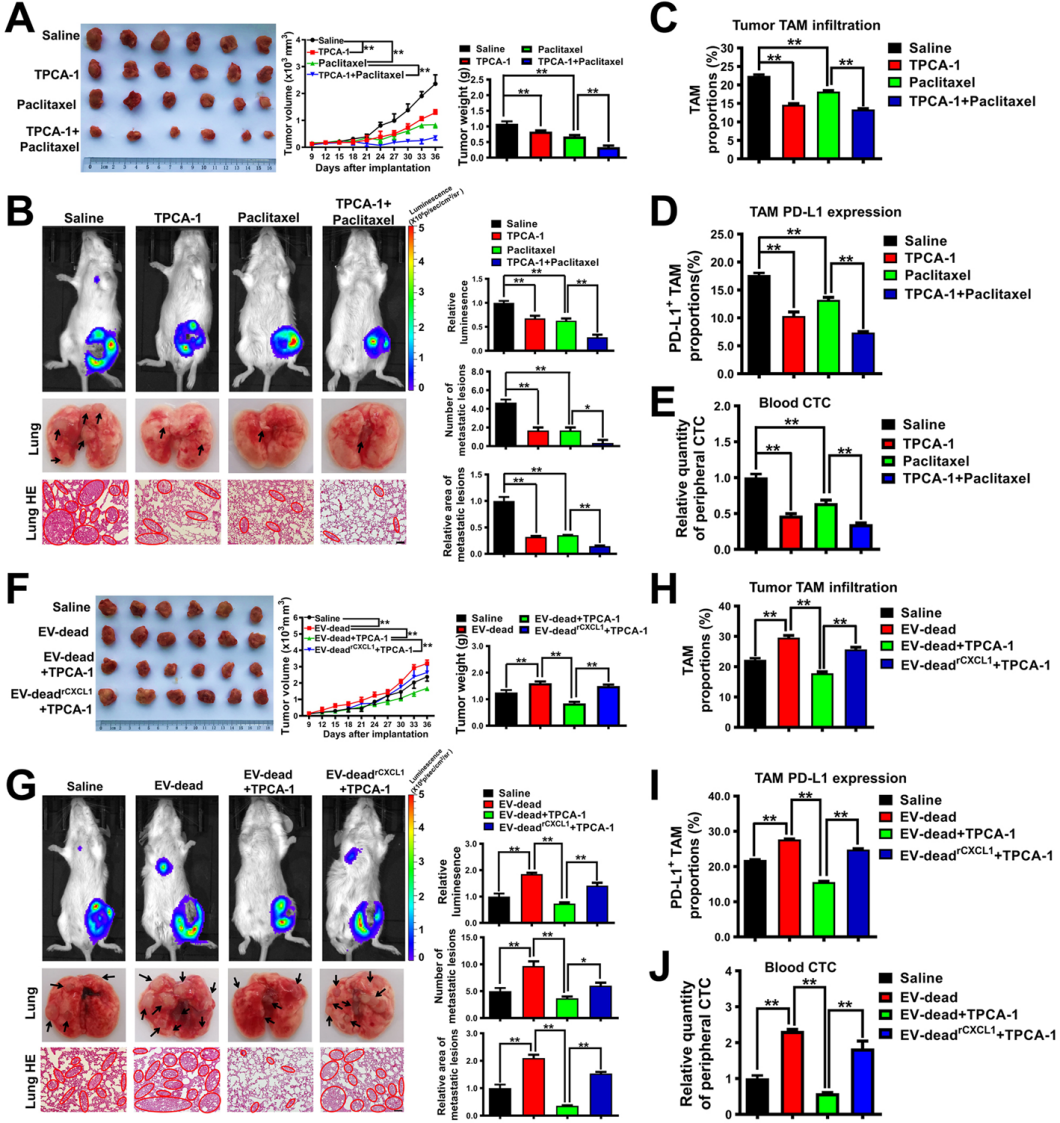
TPCA-1 chemosensitizes TNBC to paclitaxel and inhibits CXCL1^EV-dead^-induced TNBC growth and lung metastasis in vivo*.* **A** Representative images of tumors and the tumor volume curves (*n* = 6). TPCA-1 (10 mg/kg/d) and paclitaxel (10 mg/kg/3d) were administered. **B** Representative images of the in vivo imaging assay and lung HE staining assay; *n* = 3. Scale bar: 100 μm. **C**, **D** The infiltration levels of CD45^+^/F4/80^+^/CD206^+^ TAMs and CD45^+^/F4/80^+^/PD-L1^+^ TAMs in the TME of mice following treatment with TPCA-1, paclitaxel, or the combination of TPCA-1 and paclitaxel; *n* = 3. **E** The CTC quantity in the peripheral blood of mice when they were treated as indicated; *n* = 3. **F** Representative images of tumors and the tumor volume curves (*n* = 6). EV-dead and EV-dead^rCXCL1^ (200 μg/20 g weight, q3d) were administered by peritumoral injection. **G** Representative images of the in vivo imaging assay and lung HE staining assay; *n* = 3. Scale bar: 100 μm. **H**, **I** The infiltration levels of CD45^+^/F4/80^+^/CD206^+^ TAMs (*n *= 3) and CD45^+^/F4/80^+^/PD-L1^+^ TAMs (*n* = 6) in the TME of mice when they were treated as indicated. **J** The CTC quantity in the peripheral blood of mice when they were treated as indicated; *n* = 3. Data are presented as mean ± SD. ^*^p < 0.05, ^**^p < 0.01

Finally, the chemosensitizing activity of TPCA-1 was validated in vivo. As shown in Fig. 9A, B, TPCA-1 treatment (10 mg/kg/d) alone moderately inhibited TNBC growth and lung metastasis in the mouse 4 T1-Luc xenograft model. Meanwhile, TPCA-1 treatment significantly inhibited the infiltration and PD-L1 expression of TAMs in the TME (Fig. 9C, D), and decreased CTCs in the blood of 4 T1-Luc xenograft-bearing mice (Fig. 9E). Additionally, paclitaxel treatment significantly elevated EV secretion and promoted M2 phenotype macrophages (TAMs) infiltration in paclitaxel-induced necrotic regions of breast tumors (Fig. S12). More importantly, TPCA-1 could chemosensitize TNBC to paclitaxel, leading to more significant inhibitory effects on TNBC growth, lung metastasis, TAM infiltration and PD-L1 expression in the TME, and reduced CTC infiltration to the blood (Fig. 9A–E). Notably, TPCA-1 treatment at a dose of 10 mg/kg/d for 27 days exhibited no noticeable hepatotoxicity, nephrotoxicity, or hematotoxicity in vivo (Table S2), suggesting the long-term biosafety and the promising druggability of TPCA-1. We also sought to investigate whether TPCA-1 could inhibit the promotion effect of CXCL1^EV-dead^ on TNBC growth and metastasis. As shown in Fig. 9F, G, TPCA-1 treatment remarkably suppressed the induction effect of CXCL1^EV-dead^ on TNBC growth and lung metastasis in mouse 4 T1-Luc xenograft, while EV-dead^rCXCL1^ (CXCL1 overloading in EV-dead) blocked the inhibitory effect of TPCA-1. Additionally, TPCA-1 treatment also significantly inhibited the promotion effects of CXCL1^EV-dead^ on TAM infiltration and PD-L1 expression in the TME, and CTC infiltration to the blood. However, EV-dead^rCXCL1^ partially abrogated these effects, indicating that the pharmacological effect of TPCA-1 can be partially attributed to CXCL1 inhibition (Fig. 9H–J). Taken together, these results indicate that TPCA-1 not only chemosensitizes TNBC to paclitaxel but also inhibits CXCL1^EV-dead^-induced TNBC growth and lung metastasis in vivo.

## Discussion

TNBC is the most aggressive subtype of breast cancer, characterized by poor clinical outcomes and a lack of therapeutic strategies [2]. Currently, cytotoxic chemotherapy remains the standard treatment for TNBC patients. The mainstay regimens include taxane-, anthracycline-, and platinum-based chemotherapy. While chemotherapy is effective in decreasing the risk of death and distant recurrence in TNBC patients [2], emerging evidence has also indicated that chemotherapy also plays a key role in mediating cancer metastasis [6–9]. Indeed, chemotherapy has been reported to increase the infiltration of neutrophils in pancreatic cancer and results in metastasis via Gas6/AXL signaling [46]. Meanwhile, neoadjuvant chemotherapy has been shown to induce TNBC metastasis by modulating the TME [6]. Paclitaxel has also been shown to increase CTCs in breast cancer and facilitate metastatic cell seeding in the lung by upregulating the stress-inducible gene *ATF3* in nonmalignant host cells [47]. Taken together, these findings suggest that a better understanding of chemotherapy-induced metastasis will assist with the development of novel therapeutic strategies to improve cancer prognosis. In the current study, we demonstrate that paclitaxel could promote CXCL1-enriched EV-dead secretion from dying TNBC cells, which served to remodel the pro-metastatic TME by polarizing M2 macrophages through activating EED/PD-L1 signaling. More importantly, pharmacological blockage of the CXCL1^EV^ signal in TNBC cells by TPCA-1 effectively chemosensitized paclitaxel and restrained TNBC metastasis both in vitro and in vivo. Our findings highlight the important significance of dying cell-released EV signals in mediating cancer metastasis. A series of previous studies also demonstrated that dying cell-released components can induce an immunosuppressive TME. Indeed, IL-1α can be rapidly released by necrotic cells to promote malignant cell transformation and proliferation [48]. Additionally, dying cells contribute to an increase in potassium, which impairs T cell receptor signaling and limits effector T cell responses against cancer [49]. Meanwhile, emerging evidence has also suggested that EVs can participate in multiple cellular processes and contribute to cancer development; however, most previous studies have focused on the biological functions of living cancer cell-derived EVs. Indeed, Wang et al. reported that pancreatic cancer-derived exosomal miR-301a could promote pancreatic cancer metastasis by mediating M2 macrophage polarization [50]. Morrissey et al. reported that lung cancer cell-derived EVs could drive the infiltration of immunosuppressive macrophages within the pre-metastatic niche (PMN) through glycolytic dominant metabolic reprogramming [51]. The current study demonstrated that EVs derived from dying TNBC cells could significantly induce the immune escape and lung metastasis of TNBC by modulating TAMs, suggesting that dying cell-released EVs play a crucial role in facilitating the immunosuppressive TME and poor prognosis of cancer. However, whether other types of immune cells are involved in this process needs to be further studied.

In terms of molecular mechanisms, chemokine CXCL1 was found to be enriched in dying cell-released EVs. CXCL1 represents one of the most abundant chemokines in the TME, and its level in mammary tumor tissue tends to be increased compared to that in normal breast tissue. In addition, CXCL1 has been found to show higher expression levels in TNBC than other breast cancer subtypes [52, 53]. CXCL1 elevation in breast stroma usually predicts poor OS and recurrence-free survival (RFS) of patients with breast cancer [43]. CXCL1 can promote breast cancer growth and metastasis through multiple mechanisms, such as inducing epithelial-mesenchymal transformation, promoting the self-renewal of CSCs, inducing autophagy, and accelerating MDSC infiltration and PMN formation [34, 54]. Our previous study has revealed the important role of TAM-derived CXCL1 in promoting breast cancer metastasis and validated its therapeutic value [34, 55]. In this study, we demonstrated that paclitaxel and adriamycin could induce the release of CXCL1-enriched EVs from dying TNBC cells, which is consistent with the previous reports that chemotherapeutic drugs could increase CXCL1 levels in mice [56, 57]. However, in contrast to previous studies, we identified that CXCL1 mainly existed in the EVs. Meanwhile, chemotherapy-induced CXCL1^EV-dead^ signal was identified as the crucial molecular determinant for promoting macrophage M2 polarization and elevating PD-L1 expression to facilitate cancer metastasis. This finding is also consistent with the existing report in Nature showing that chemotherapy-induced necroptosis CXCL1 signals could promote pancreatic oncogenesis and progression by inducing adaptive immune suppression in the TME through activating PD-L1 expression on TAMs [58].

*PD*-*L1* signaling is an important mechanism utilized by immunosuppressive TAMs to inhibit anticancer responses. Emerging reports have suggested that TAMs represent the major cellular source for maintaining PD-L1 expression in the TME in multiple tumors, including metastatic breast cancer, and that PD-L1 is crucial for the activation and M2 polarization of macrophages [44, 45]. Notably, TNBC is particularly characterized by increased infiltration of TAMs and expression of PD-L1, both of which are strongly correlated with poor prognosis of TNBC [59, 60]. It has been reported that nearly half of TNBC patients were PD-L1 positive, and RNA profiles of PD-L1 positive TNBC samples suggested increased TAM infiltration features [61]. TAMs and PD-L1 have emerged as promising immunotherapy targets for TNBC. Therefore, molecular elucidation of PD-L1 regulation in TAMs is urgently needed for the successful development of treatment strategies and targeting agents to inhibit TNBC immune escape. It has been well known that CXCL1 could recruit immune cells and activate their intracellular signal transduction by binding to its receptor CXCR2 [34, 58]. However, EV uptake by macrophages is mainly mediated by phagocytosis or endocytosis [62]. After phagocytosis, the cargo molecule of CXCL1 in EV-dead was released into the cytoplasm. Therefore, we speculate that CXCR2 may not be involved in EV-dead^CXCL1^-induced PD-L1 expression and M2 phenotype polarization of macrophages. Therefore, we focused on identifying the downstream molecules that participate in CXCL1-induced PD-L1 expression in TAMs. It was found that CXCL1 induced the expression and nuclear translocation of EED in macrophages, which bound to the 5′-GTTCCACTC-3′ region of the *PD-L1* promoter and transcriptionally elevated *PD-L1* expression. This finding was consistent with the existing reports demonstrating that CXCL1 promoted PD-L1 expression in glioblastoma multiforme cells [63] and hepatic cells [64] by increasing the activity of the *PD-L1* promoter. Meanwhile, our results also suggest that the combination of PD-L1 blockade and paclitaxel may achieve synergistic inhibition of TNBC. Indeed, PD-L1 expression in macrophages is associated with the response to neoadjuvant chemotherapy in TNBC [65]. Meanwhile, PD-L1 expression in residual mammary tumors has been suggested as a prognostic marker in the non-pathological complete response (pCR) patients after receiving neoadjuvant chemotherapy [66]. Notably, recent advancements in the therapeutic landscape of TNBC have been marked by the efficacious integration of PD-L1 inhibitors with conventional chemotherapy. For example, in the IMpassion 130 clinical investigation, atezolizumab in conjunction with nab-paclitaxel demonstrated a notable improvement in both progression-free survival (PFS) and OS among TNBC patients [67, 68]. These results promoted the approval of atezolizumab plus nab-paclitaxel as a first-line therapeutic option for PD-L1 positive unresectable locally advanced or metastatic TNBC by the FDA. Additionally, the KEYNOTE-522 clinical trial revealed that the combination of pembrolizumab with chemotherapy significantly elevated pCR rates in TNBC patients compared to chemotherapy alone [69]. This strategy has been approved by the FDA for the treatment of high-risk, early-stage TNBC, as well as metastatic TNBC patients with high PD-L1 expression. Furthermore, emerging neoadjuvant clinical trials have also demonstrated the encouraging outcomes of atezolizumab plus chemotherapy in non-small cell lung cancer (NSCLC) [70, 71]. Our study elucidated that PD-L1 blockage abrogated the induction effect of chemotherapy-elicited CXCL1^EV-dead^ signal on the proliferation and invasion of the co-cultured TNBC cells both in vitro and in vivo. This finding provides an experimental basis for the above combination regimen of chemotherapy with PD-L1 inhibitors in TNBC and NSCLC patients. As chemotherapy-induced CXCL1 was identified as an upstream regulator of PD-L1 in this study, future clinical studies regarding CXCL1 inhibitors plus neoadjuvant chemotherapy are worth investigating in the future.

Based on the above results, we speculated that the selective inhibition of CXCL1^EV-dead^ signaling by small molecule inhibitors may be a promising treatment strategy to enhance chemoresponse and inhibit CXCL1^EV-dead^-induced TNBC metastasis. In this study, TPCA-1 exhibited the strongest inhibitory activity on CXCL1 secretion among 80 compounds from the chemokine inhibitor library. TPCA-1 is a potent and selective inhibitor of IKK-2 with an IC_50_ of 17.9 nM. TPCA-1 functions by inhibiting the release of inflammatory cytokines (e.g., IL-1β, IL-6, IL-8 and TNF-α) and inactivating NF-κB and STAT pathways [72]. The anti-inflammatory properties of TPCA-1 have been well-identified, and recent studies have focused on using TPCA-1 as a core encapsulating drug to prepare anti-inflammatory nanocarriers [73]. In the present study, TPCA-1 significantly decreased CXCL1 cargo in EV-dead. Notably, it has been reported that IL-1β and NF-κB pathways control *CXCL1* gene transcription [74], whereas blocking the IL-1β and NF-κB signaling can alter the secretion of CXCL1 [75]. These results suggested that the inhibitory effect of TPCA-1 on CXCL1 expression may be partially mediated through suppressing IL-1β and NF-κB pathways. This may also be the reason why TCPA-1 alone is effective in inhibiting breast tumor growth and metastasis in this study. Recently, TPCA-1 has attracted increasing attention, and preclinical studies have proven the therapeutic efficacy of TPCA-1 in autoimmune and inflammatory diseases, such as rheumatoid arthritis, periodontitis, rhinitis, and pneumonia [72]. However, existing reports on the anti-cancer activity and mechanisms of TPCA-1 have been limited until now. Nan et al. reported that TPCA-1 could inhibit mutant EGFR-associated human NSCLC by inactivating the STAT3 and NF-κB pathways [76]. Furthermore, comprehensive pan-cancer analysis identifies cellular senescence as a new cancer therapeutic target. Interestingly, the cellular senescence pathways in multiple malignancies were sensitive to TPCA-1 [77]. Moreover, TPCA-1 also synergistically restrained the growth, migration, and invasion of medulloblastoma cells by blocking the Sonic hedgehog and NF-κB pathways [78]. Using transcriptome-based drug repositioning, TPCA-1 was also screened as a potential selective inhibitor of esophagus squamous carcinoma [79]. Here, we report for the first time that TPCA-1 could significantly chemosensitize paclitaxel and inhibit CXCL1^EV-dead^-induced growth and metastasis of TNBC. Notably, the anti-cancer effect of TPCA-1 was partially attributed to CXCL1 inhibition since CXCL1 overexpressing EV-dead (EV-dead^rCXCL1^) reduced the inhibitory effect of TPCA-1 on EV-dead-induced breast tumor growth and metastasis. Our results highlight the development value of TPCA-1 in inhibiting dying-cell-released cytokines during chemotherapy. More importantly, TPCA-1 showed limited hepatotoxicity, nephrotoxicity, and hematotoxicity in vivo*,* suggesting that it may be safely used along with chemotherapy. These results suggest that TPCA-1 may be developed as an adjuvant agent co-administrated with chemotherapy to clean dying cell-released signals and improve prognosis. However, in-depth pre-clinical and clinical studies are still required to validate the value of TPCA-1 in cancer therapy and examine its druggability.

## Conclusion

Taken together, our study demonstrated that dying TNBC cells induced by paclitaxel could secrete CXCL1-enriched EVs to promote TNBC growth and metastasis by activating TAM/PD-L1 signaling. Furthermore, we demonstrated that TPCA-1 could inhibit CXCL1^EV-dead^ signals and chemosensitize TNBC to improve prognosis. Our findings not only delineate the novel biological mechanism of CXCL1^EV-dead^/TAM/PD-L1 signaling in dying cell-induced immunosuppressive TME but also highlight the potential use of TPCA-1 as a CXCL1^EV^ inhibitor to chemosensitize TNBC and limit dying cell signal-induced metastasis.

### Supplementary Information

**Supplementary Material 1.**

## Data Availability

Data are available on reasonable request. All data generated or analyzed during this study are included either in the article or in the supplementary files.

## Abbreviations

TNBC: Triple-negative breast cancer
EV-dead: Extracellular vesicles from apoptotic TNBC cells
TPCA-1: 2-[(aminocarbonyl) amino]-5-(4-fluorophenyl)-3-thiophenecarboxamide
EV-alive: EVs of untreated TNBC cells
OS: Overall survival
TME: Tumor microenvironment
DAMPs: Damage-associated pattern molecules
DCs: Dendritic cells
TAMs: Tumor-associated macrophages
EVs: Extracellular vesicles
MDSC: Myeloid-derived suppressor cell
Tregs: Regulatory T cells
PMA: Phorbol-12-myristate-13-acetate
ADR: Adriamycin
mAb: Monoclonal antibody
BCSCs: Breast cancer stem cells
CM: Conditioned medium
CTC: Circulating tumor cell
CHIP: Chromatin immunoprecipitation
EV-dead^ADR^: ADR elicited EV-dead
NA: Neutralizing antibody
DEPs: Deferentially expressed proteins
TFs: Transcription factors
EED: Embryonic ectoderm development protein
EV-dead^rCXCL1^: CXCL1 overloading in EV-dead
PMN: Pre-metastatic niche
RFS: Recurrence-free survival
PFS: Progression-free survival
NSCLC: Non-small cell lung cancer
