# Supplementary material for: Chemotherapy-elicited extracellular vesicle CXCL1 from dying cells promotes triple-negative breast cancer metastasis by activating TAM/PD-L1 signaling
Source: J Exp Clin Cancer Res. 2024 Apr 23;43:121. doi: 10.1186/s13046-024-03050-7 (PMC11036662; doi:10.1186/s13046-024-03050-7)
Supplement: Supplementary file 1 — Supplementary Material 1. [file 13046_2024_3050_MOESM1_ESM.docx]

**Supplementary Figures and Tables**

**
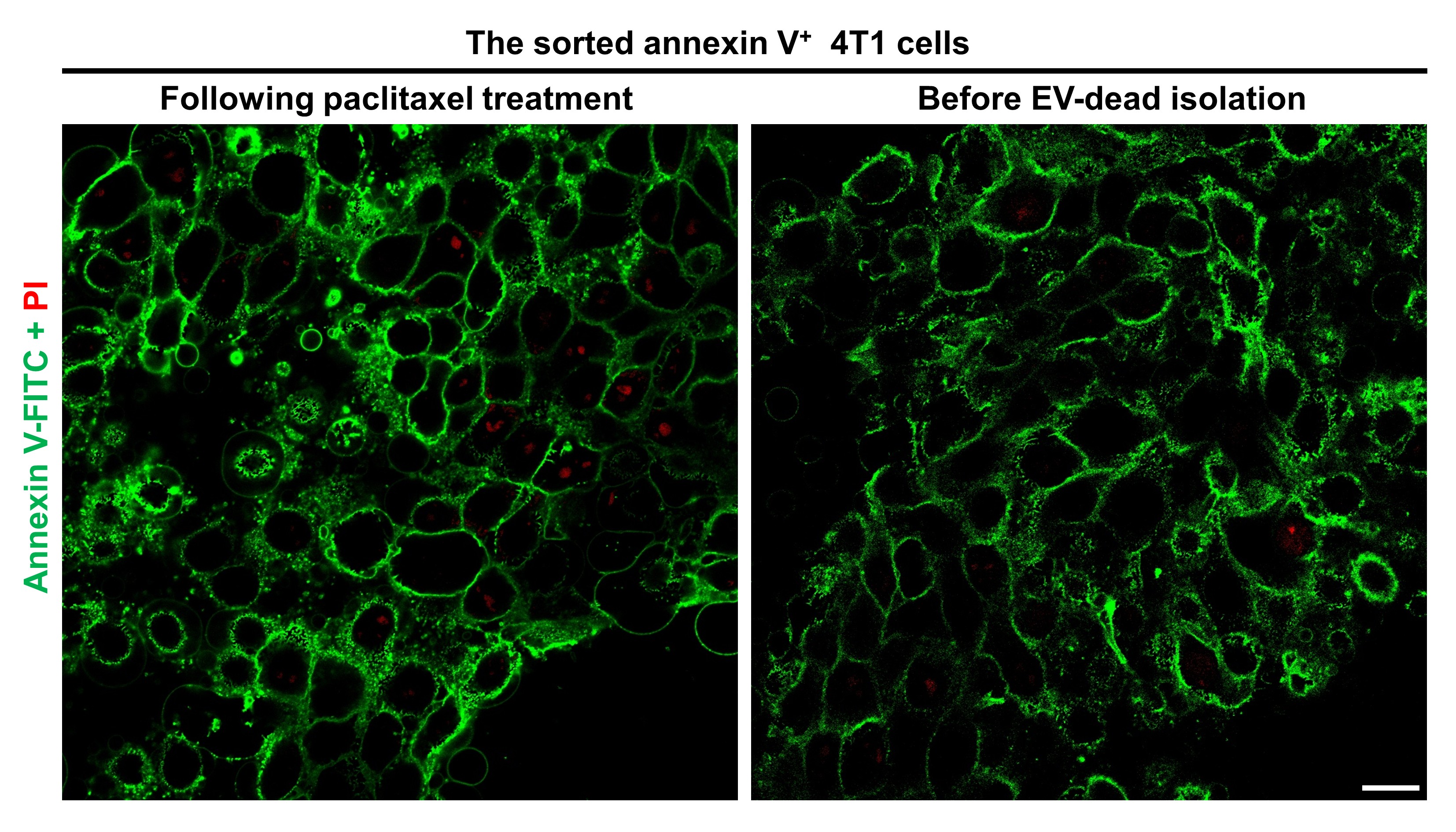
**

**Figure S1. Apoptotic status of the sorted 4T1 cells following paclitaxel treatment and before EV-dead isolation*.*** Cells were stained with annexin V-FITC/PI and observed under a fluorescent microscope. Scale bar: 20 μm. n = 3.


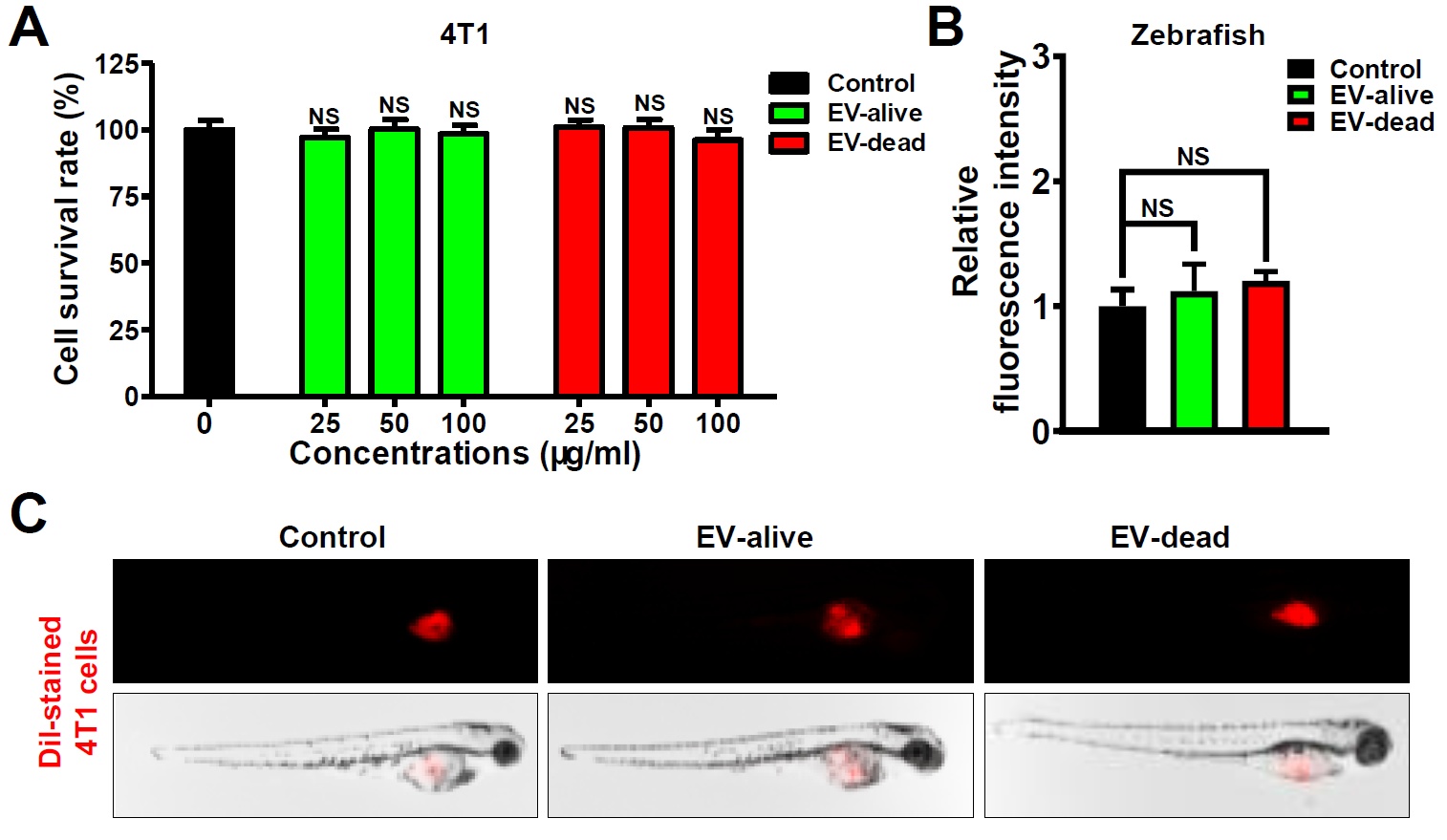


**Figure S2. Effects of EV-alive and EV-dead on the proliferation of TNBC 4T1 cells*.* (A)** The viability of 4T1 cells after EV-alive and EV-dead treatment for 48 h was investigated using CCK-8 assay *in vitro* (n = 8). **(B–C)** The effects of EV-alive (100 μg/ml) and EV-dead (100 μg/ml) on the proliferation and metastasis of 4T1 cells in zebrafish (n = 6). Data are presented as mean ± SD.

**
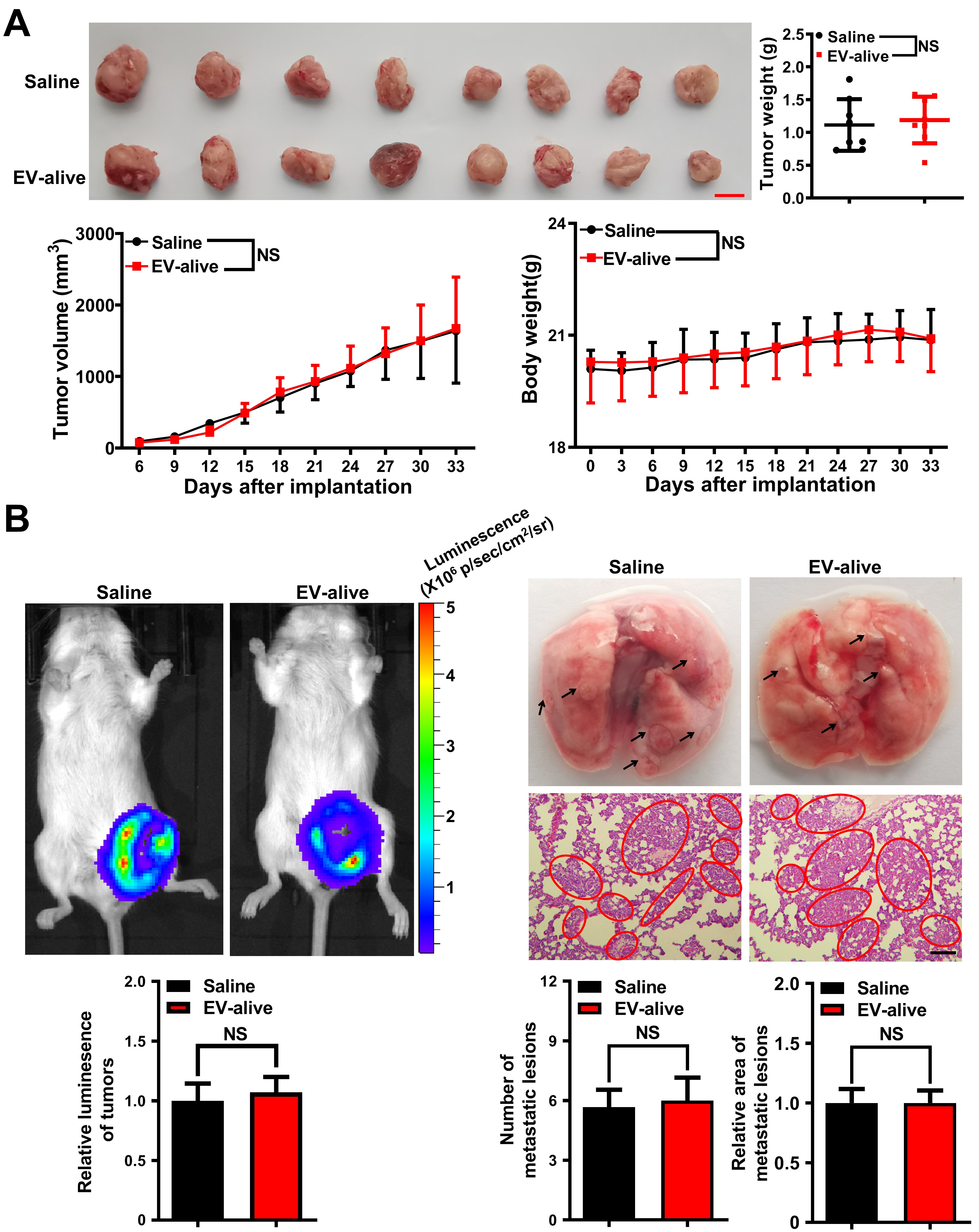
**

**Figure S3. EV-alive treatment exhibited little effect on the growth and lung metastasis of 4T1-Luc xenografts. (A)** Peritumoral injection with EV-alive (200 μg/20 g weight, q3d) exhibited little significant effect on tumor volume, tumor weight, and body weight; n = 8. Scale bar: 1 cm. **(B)** Representative pictures of the *in vivo* imaging assay, lungs and the lung HE staining assay; Scale bar: 100 μm; n = 3. Data are presented as mean ± SD.


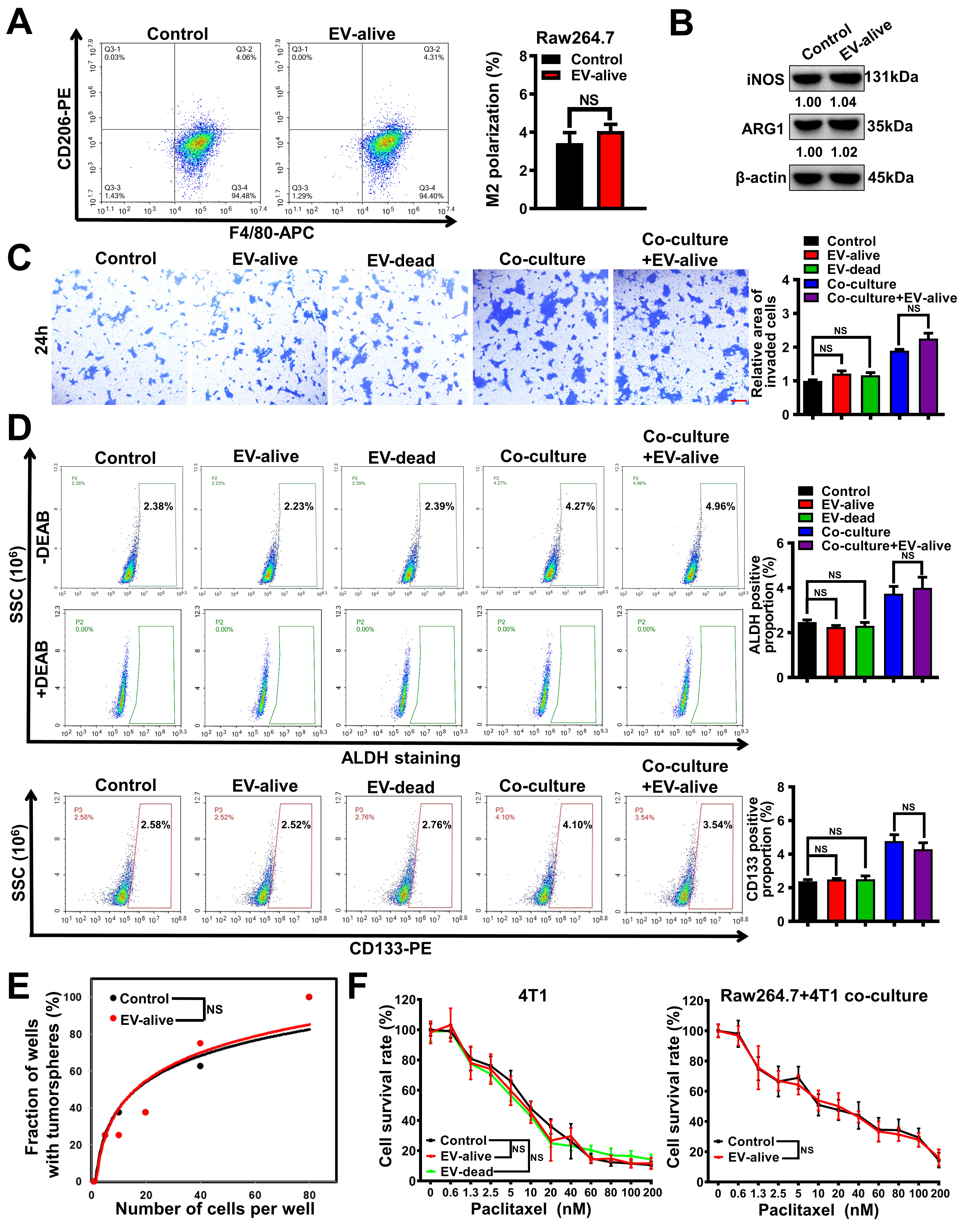


**Figure S4. Effects of mouse EV-alive and EV-dead on the invasion and BCSC subpopulation of 4T1 cells in the presence or absence of Raw264.7 macrophage co-culture. (A–B)** Polarization changes of Raw264.7 macrophages after EV-alive treatment (100 μg/ml) for 48 h; n = 3. **(C)** Invasion efficacy changes of the individually cultured or co-cultured 4T1 cells after EV treatment as indicated for 24 h; Scale bar: 100 μm; n = 3. **(D)** The ALDH^+^ BCSC subpopulation and the CD133^+^ BCSC subpopulation in the individually cultured or co-cultured 4T1 cells after EV treatment as indicated for 48 h; n = 3. **(E)** The self-renewal activity of mouse BCSCs after treatment with the CM of EV-alive-treated Raw264.7 cells; n = 8. **(F)** Chemotherapeutic sensitivity of the individually cultured or co-cultured 4T1 cells after EV treatment as indicated for 48 h; n = 8. EV-alive and EV-dead were used at 100 μg/ml. Data are presented as mean ± SD.

**
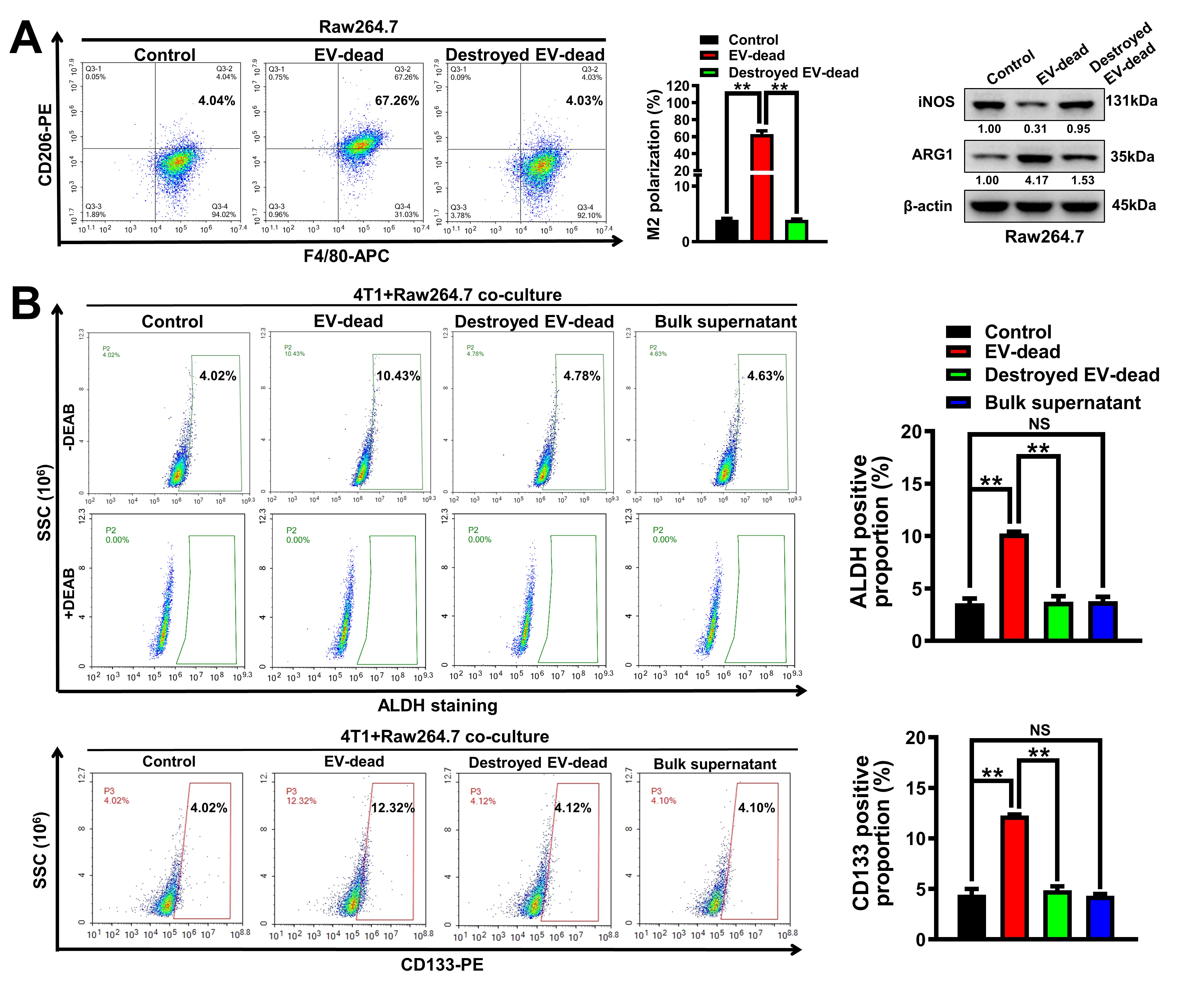
**

**Figure S5. Mouse EV-dead** **destruction abrogated the induction effect of EV-dead on the M2 polarization of Raw264.7 macrophages and the self-renewal of the co-cultured 4T1 cells. (A)** Polarization changes of Raw264.7 macrophages after treatments with EV-dead, destroyed EV-dead or bulk supernatant for 48 h; 1% SDS was used to destroy the EV membranes; EV concentration, 50 μg/ml. **(B)** The ALDH^+^ BCSC subpopulation and CD133^+^ BCSC subpopulation in the co-cultured 4T1 cells after treatments as indicated for 48 h; EV concentration, 50 μg/ml. n = 3. Data are presented as mean ± SD. ^**^*p* < 0.01.


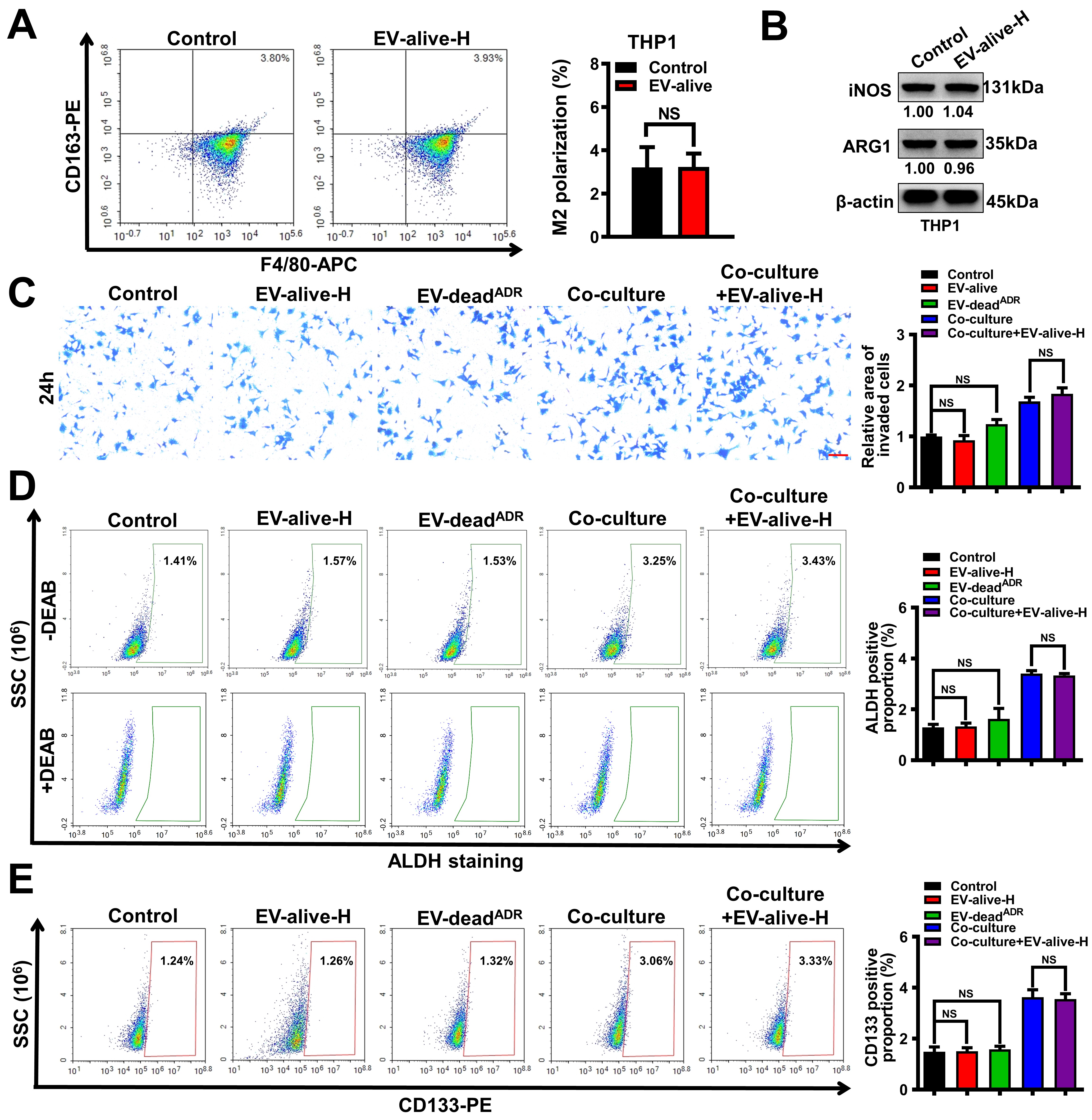


**Figure S6. Effects of human EV-alive and EV-dead on the invasion and BCSC subpopulation of MDA-MB-231 cells in the presence or absence of THP1 macrophage co-culture. (A–B)** Polarization changes of THP1 macrophages after EV-alive-H treatment (100 μg/ml) for 48 h; **(C)** Invasion efficacy changes of the individually cultured or co-cultured MDA-MB-231 cells after EV treatment as indicated for 24 h; Scale bar: 100 μm; **(D–E)** The ALDH^+^ BCSC subpopulation and the CD133^+^ BCSC subpopulation in the individually cultured or co-cultured MDA-MB-231 cells after EV treatment as indicated for 48 h; EV-alive-H and EV-dead^ADR^ were used at 100 μg/ml. n = 3. Data are presented as mean ± SD.

**
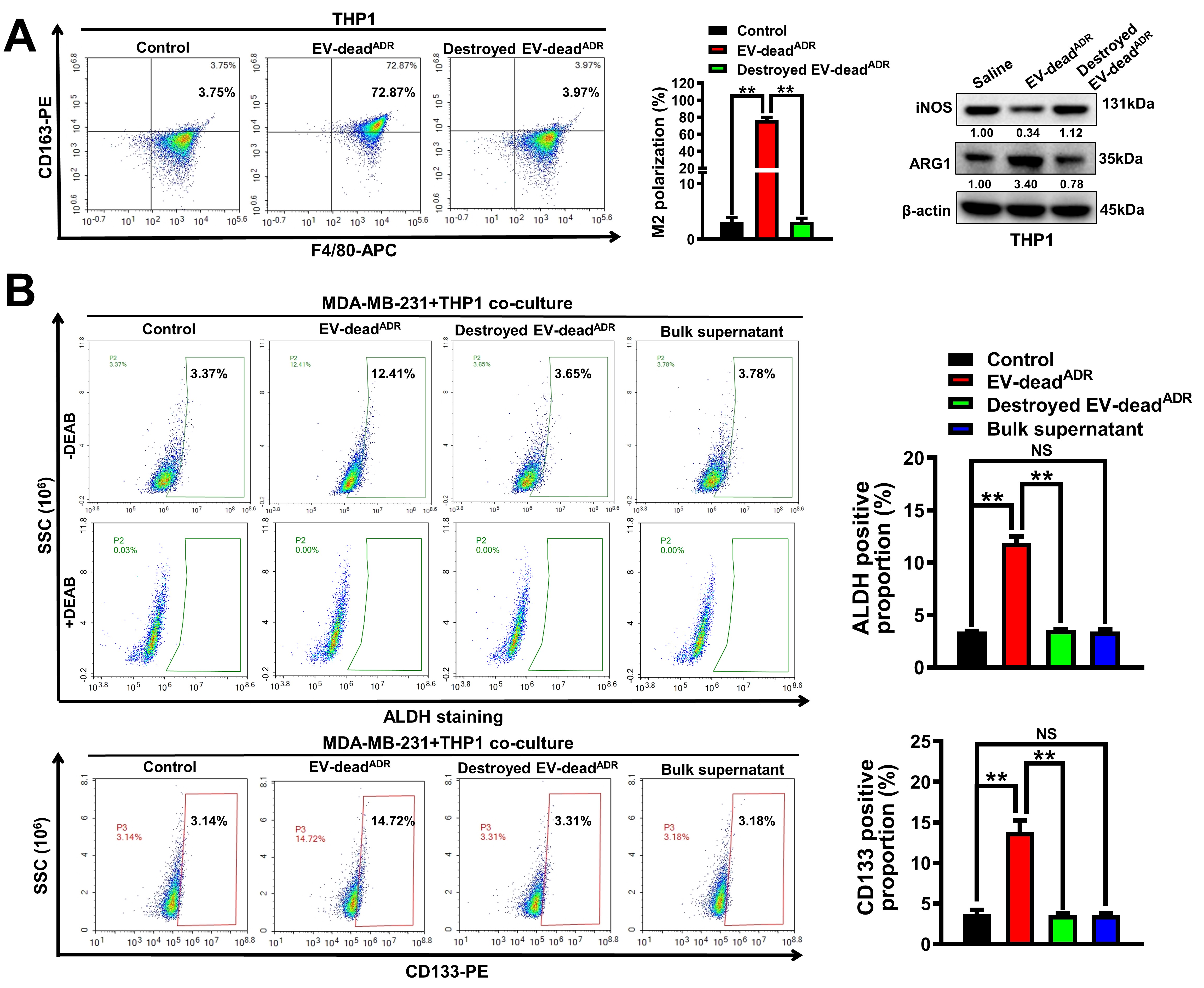
**

**Figure S7. Human EV-dead** **destruction abrogated the induction effect of EV-dead on the M2 polarization of THP1 macrophages and the self-renewal of the co-cultured MDA-MB-231 cells. (A)** Polarization changes of THP1 macrophages after treatments with EV-dead^ADR^ (Human EV-dead), destroyed EV-dead^ADR^ or bulk supernatant for 48 h; 1% SDS was used to destroy the EV membranes; EV concentration, 50 μg/ml. **(B)** The ALDH^+^ BCSC subpopulation and CD133^+^ BCSC subpopulation in the co-cultured MDA-MB-231 cells after treatments as indicated for 48 h; EV concentration, 50 μg/ml. Data are presented as mean ± SD. ^**^*p* < 0.01.


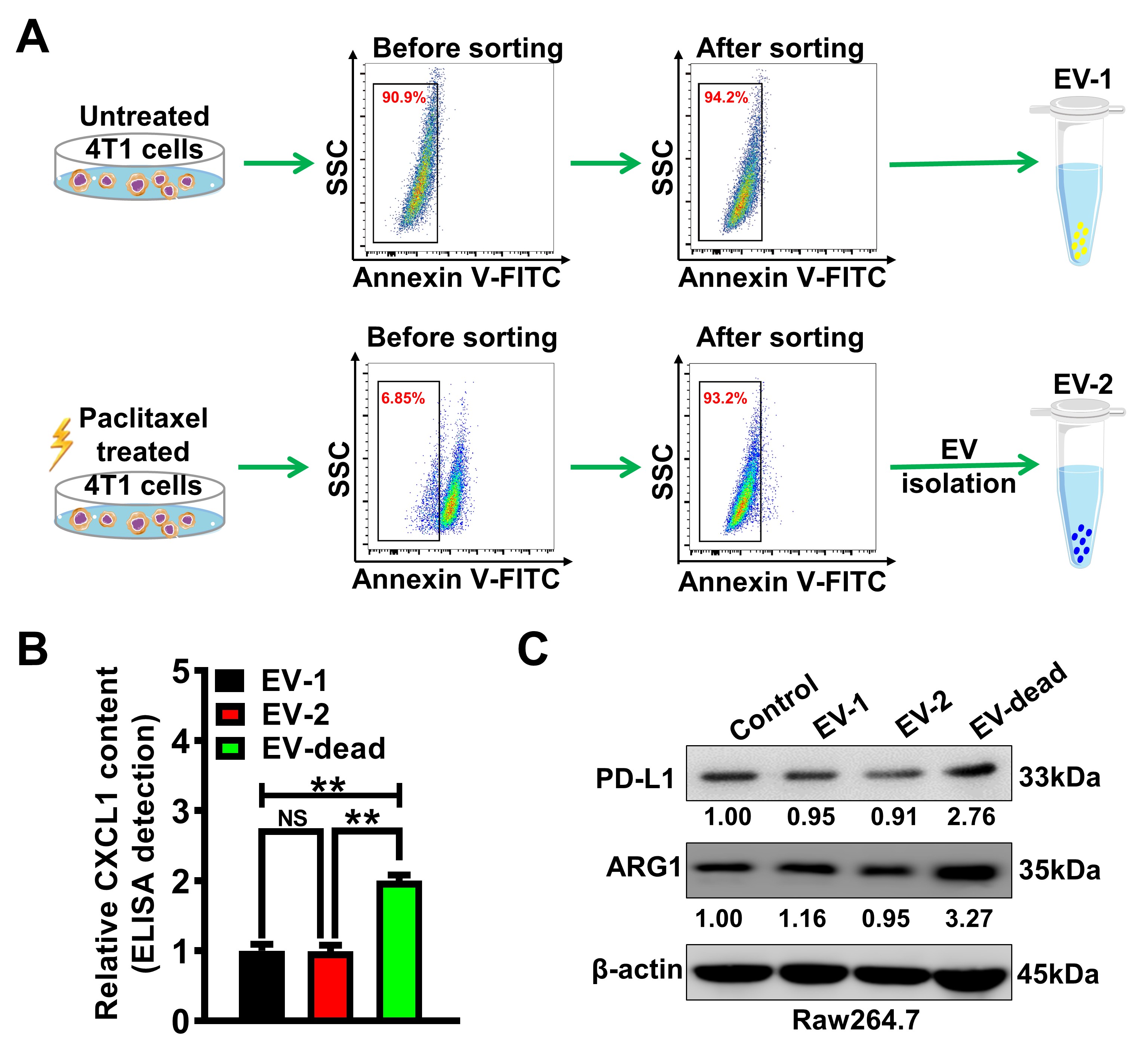


**Figure S8. EVs derived from the annexin V^-^ 4T1 cells were unable to activate TAM/PD-L1 signaling. (A)** A schematic diagram of EV isolation from the conditioned medium of the annexin V^-^ 4T1 cells. Annexin V^-^ 4T1 cells were sorted from both untreated 4T1 cells and paclitaxel-treated 4T1 cells. 4T1 cells were treated with 1 μM paclitaxel for 24 h. **(B)** CXCL1 concentrations in different EVs were quantified by ELISA assay. **(C)** Effects of different EVs (50 μg/ml) on TAM/PD-L1 activation were investigated by detecting PD-L1 and ARG1 expression changes in Raw264.7 macrophages. Raw264.7 cells were treated as indicated for 48 h. n = 3. Data are presented as mean ± SD. ^**^*p* < 0.01.


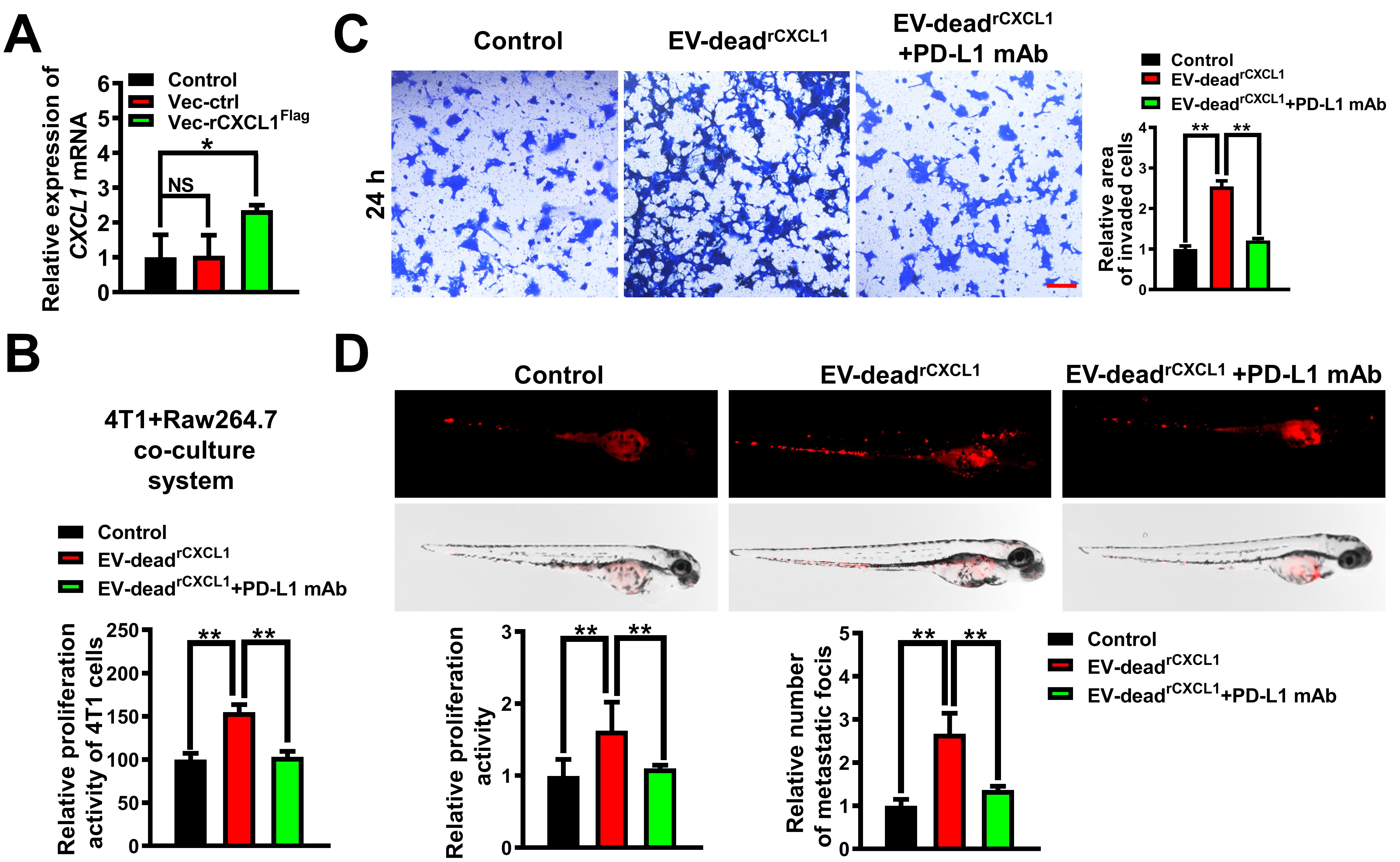


**Figure S9. PD-L1 blockage partially abrogated the induction of CXCL1^EV-dead^ on the proliferation and metastasis of the co-cultured/co-injected 4T1 cells. (A)** The successful generation of 4T1/rCXCL1^Flag^ cells was validated by QPCR assay; n = 3. **(B–C)** The proliferation and invasion activity changes of the co-cultured 4T1 cells after treatment as indicated; Scale bar: 100 μm. n = 3. **(D)** The proliferation and metastasis activity changes of the co-injected 4T1 cells in the zebrafish breast cancer xenotransplantation model after treatment as indicated; Anti-PD-L1 mAb, 10μg/ml; EV-dead^rCXCL1^, 50μg/ml; n = 6. Data are presented as mean ± SD. ^*^*p* < 0.05, ^**^*p* < 0.01.


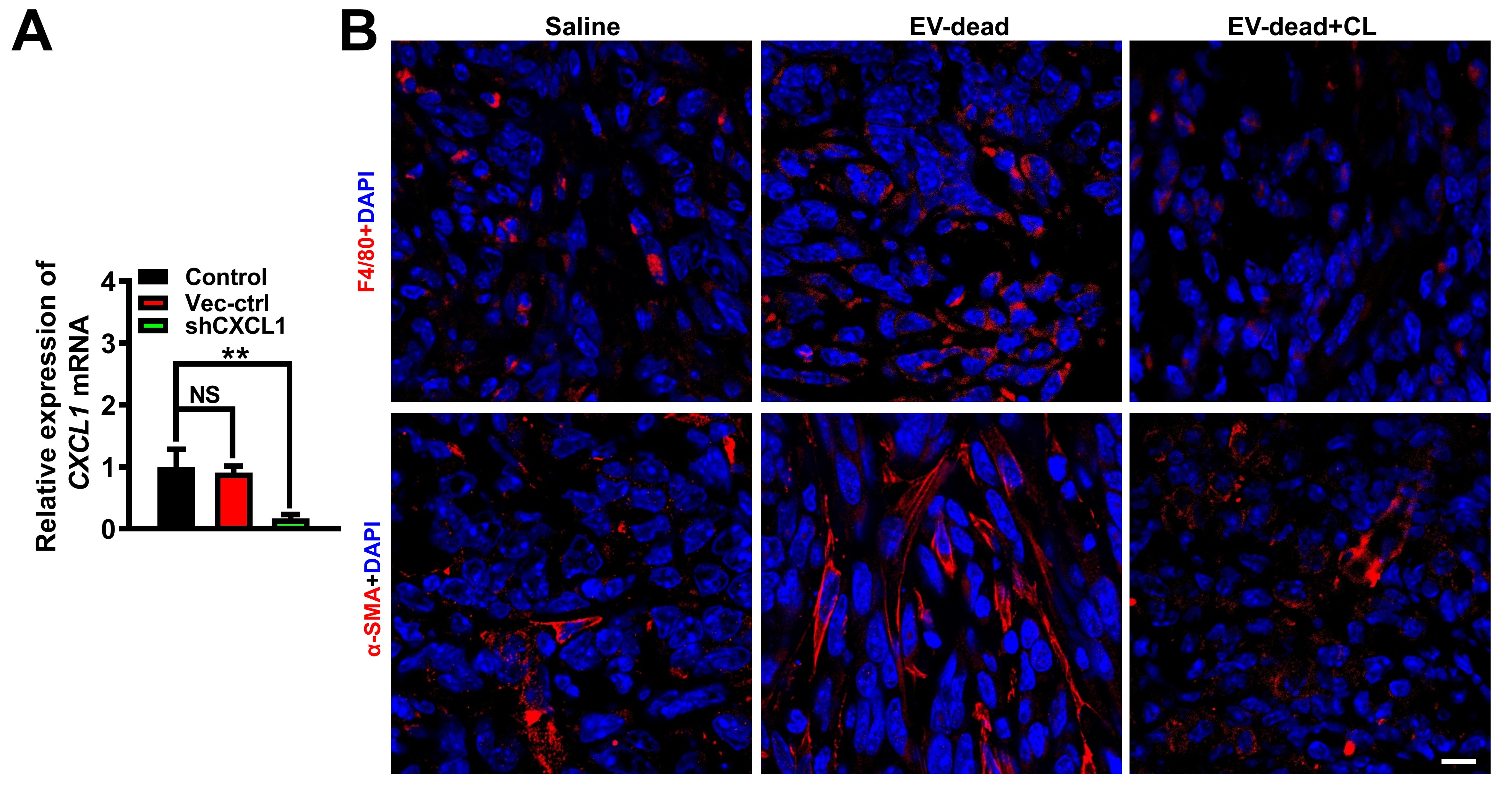


**Figure S10. Effect of CL on macrophages and fibroblasts. (A)** The successful knockdown of CXCL1 in 4T1 cells was validated by QPCR assay. **(B)** The expression levels of F4/80 (macrophage marker) and α-SMA (fibroblast marker) in the TME of breast tumors were detected by an immunofluorescence assay. Scale bar: 10 μm. n = 3. Data are presented as mean ± SD. ^**^*p* < 0.01.


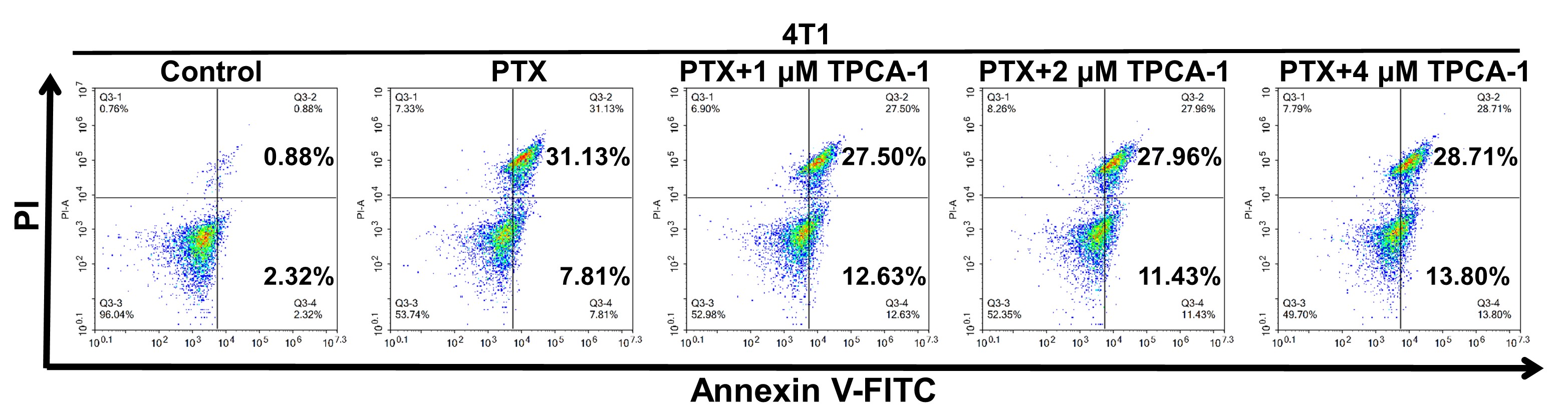


**Figure S11. Effects of different concentrations of TPCA-1 on PTX-induced apoptosis of 4T1 cells.** 4T1 cells were cultured alone and treated with PTX (50 nM), or the combination of PTX and TPCA-1 (1–4 μM) for 48 h. Apoptosis rates of 4T1 cells were detected by annexin V-FITC/PI staining and flow cytometry. Please refer to Figure 8D for the statistical analysis. n = 3.


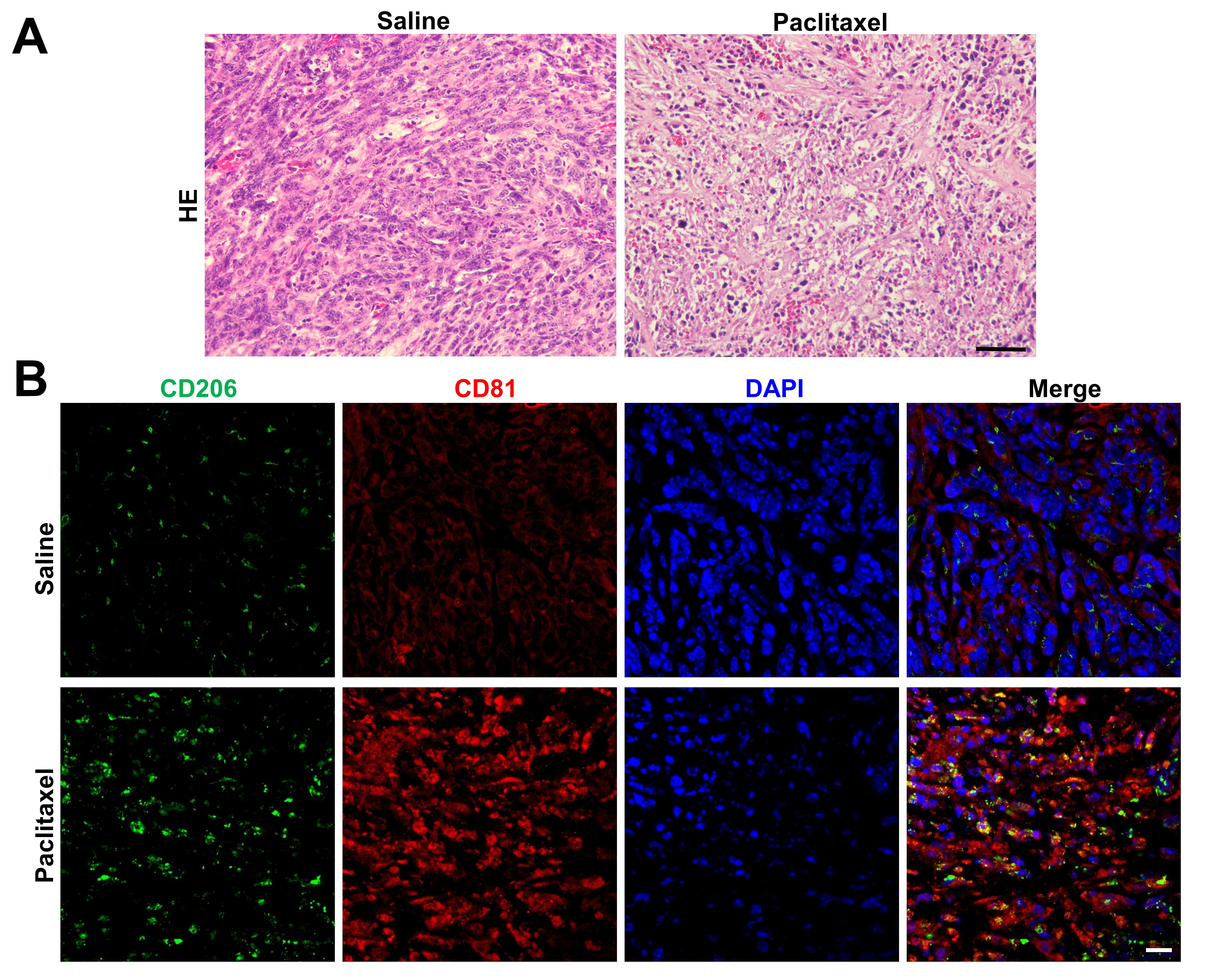


**Figure S12. Effects of paclitaxel on EV secretion and TAM infiltration. (A)** The necrotic regions in breast tumor tissues were identified by HE staining. Scale bar: 50 μm. **(B)** The expression levels of CD206 (TAM marker) and CD81 (EV marker) in paclitaxel-induced necrotic regions of breast tumors. Scale bar: 10 μm. n = 3.

| Table S1. The inhibitory effects of 80 kinds of small molecules on CXCL1 secretion from 4T1 cells. (n = 6) | | | | | |
| --- | --- | --- | --- | --- | --- |
| Rank | **Compound** | **CXCL1 secretion (% of control）** | **Rank** | **Compound** | **CXCL1 secretion (% of control）** |
| 1 | Plerixafor | 54.47±2.52 | 41 | Baricitinib Phosphate | 112.05±6.94 |
| 2 | TPCA-1 | 67.26±4.96 | 42 | PF-4136309 | 112.06±5.54 |
| 3 | C-DIM12 | 68.92±2.22 | 43 | AMD 3100 octahydrochloride | 112.37±16.00 |
| 4 | AS-604850 | 73.79±1.33 | 44 | ETC-1002 | 112.56±16.80 |
| 5 | Fudosteine | 78.01±1.80 | 45 | Camostat mesilate | 112.66±9.16 |
| 6 | ML130 (Nodinitib-1) | 78.76±17.61 | 46 | MSX-130 | 112.66±9.95 |
| 7 | Dexibuprofen | 79.12±6.46 | 47 | BX-471 | 112.87±8.97 |
| 8 | GW3965 HCl | 82.93±8.08 | 48 | Baohuoside I | 114.10±6.48 |
| 9 | BMS-309403 | 82.95±1.29 | 49 | Clarithromycin | 114.11±2.46 |
| 10 | Pyrrolidinedithiocarbamate ammonium | 83.77±10.66 | 50 | Vicriviroc maleate | 114.96±12.02 |
| 11 | BMS-813160 | 84.08±4.19 | 51 | Diacerein | 115.63±8.34 |
| 12 | Baricitinib | 85.83±4.67 | 52 | AS-252424 | 116.77±20.33 |
| 13 | GS-9620 | 87.09±11.89 | 53 | NSC5844 | 116.90±5.00 |
| 14 | Repertaxin | 89.14±6.81 | 54 | Nicotinamide-N-oxide | 118.78±5.27 |
| 15 | MSX-122 | 90.52±1.12 | 55 | JNJ-38877605 | 119.31±10.31 |
| 16 | Buflomedil hydrochloride | 94.54±7.50 | 56 | GW 501516 | 124.29±13.88 |
| 17 | Felodipine | 95.01±3.18 | 57 | C29 | 124.59±19.5 |
| 18 | Rosuvastatin calcium | 95.44±3.25 | 58 | PRT062607 hydrochloride | 125.01±11.42 |
| 19 | SRT3109 | 96.5±8.15 | 59 | WZ811 | 127.44±5.79 |
| 20 | MSX-127 | 97.07±19.34 | 60 | Azithromycin | 129.22±15.52 |
| 21 | Dextromethorphan HBr monohydrate | 97.59±4.12 | 61 | Turofexorate Isopropyl (XL335) | 129.93±4.81 |
| 22 | MRS 2578 | 98.75±24.01 | 62 | Chondroitine sulfate | 130.28±3.50 |
| 23 | Loratadine | 100.07±8.89 | 63 | Irbesartan | 133.76±4.21 |
| 24 | Telotristat Etiprate (LX 1606 Hippurate) | 100.23±2.12 | 64 | AN-2728 | 136.05±7.71 |
| 25 | Maraviroc | 100.47±11.35 | 65 | AZD2098 | 137.06±8.70 |
| 26 | Budesonide | 101.11±5.17 | 66 | AX-024 HCl | 137.59±26.57 |
| 27 | Niflumic acid | 101.32±6.40 | 67 | Arteether | 137.62±1.77 |
| 28 | ZK 756326 | 102.14±11.48 | 68 | MCC950 | 137.70±7.49 |
| 29 | Oroxylin A | 102.66±13.51 | 69 | Bindarit | 140.03±5.33 |
| 30 | Azelastine hydrochloride | 104.41±6.64 | 70 | VX-745 | 140.83±8.27 |
| 31 | R406 | 104.79±12.64 | 71 | Nadifloxacin | 148.89±6.98 |
| 32 | Rifaximin | 106.81±7.08 | 72 | IRAK-1/4 Inhibitor | 150.94±6.83 |
| 33 | UNBS-5162 | 107.24±8.98 | 73 | AS-605240 | 157.06±29.8 |
| 34 | Suplatast tosilate | 107.77±1.85 | 74 | (±)-Shikonin | 162.68±8.50 |
| 35 | DAMGO TFA (78123-71-4) | 108.68±19.13 | 75 | SC-514 | 163.65±9.27 |
| 36 | Etodolac | 109.27±4.41 | 76 | Triamcinolone | 168.95±3.98 |
| 37 | Fenofibric acid | 109.29±9.06 | 77 | SB225002 | 187.93±12.44 |
| 38 | Geniposide | 110.69±5.50 | 78 | MG-132 | 218.61±2.82 |
| 39 | Roflumilast N-oxide | 110.89±3.86 | 79 | Fostamatinib (R788) | 229.47±3.69 |
| 40 | Proglumide | 111.75±5.41 | 80 | Loxiglumide | 289.87±5.27 |

**Table S2. TPCA-1 exhibited little hepatotoxicity, nephrotoxicity, or hematotoxicity *in vivo*.**

| [**Blood**](E:/360Downloads/Dict/8.3.1.0/resultui/html/index.html#/javascript:;) [**biochemical**](E:/360Downloads/Dict/8.3.1.0/resultui/html/index.html#/javascript:;) **values** | **Saline** | **TPCA-1** | ***P* values** |  |
| --- | --- | --- | --- | --- |
| **ALT**（U/L） | 8.7 ± 0.6 | 7.3 ± 1.2 | 0.15 |  |
| **AST**（U/L） | 89.3 ±1.2 | 90.0 ± 1.0 | 0.49 |  |
| **Urea**（mmol/L） | 2.7 ±0.1 | 2.5 ± 0.2 | 0.16 |  |
| **Creatinine**（μmol/L） | 11.3 ± 0.6 | 10.7 ± 1.2 | 0.42 |  |
| **Uric acid**（μmol/L） | 179.7 ± 0.6 | 179.3 ± 0.6 | 0.52 |  |
| **WBC**（10^9/L） | 2.9 ± 0.1 | 2.5 ± 0.3 | 0.09 | |
| **RBC**（10^12/L） | 9.4 ± 0.1 | 9.8 ± 0.5 | 0.38 | |
| **Hb**（g/L） | 140.7 ± 0.6 | 150.0 ± 12.1 | 0.31 | |

ALT, Alanine transaminase; AST, Aspartate aminotransferase; WBC, White blood cell; RBC, Red blood cell; Hb, Hemoglobin. n = 3.
